# Bridging global context and local precision using a disagreement-based region specific ensemble of Swin UNETR and SegResNet for 3D glioma segmentation

**DOI:** 10.3389/frai.2026.1812932

**Published:** 2026-08-18

**Authors:** Amrit Baskota, Shubham Ghimire, Baskaran Periyasamy

**Affiliations:** School of Computer Science and Engineering (SCOPE), Vellore Institute of Technology, Vellore, Tamil Nadu, India

**Keywords:** 3D medical image segmentation, brain tumor, BRATS, convolutional neural networks (CNN), deep learning ensemble, disagreement based fusion, neuro-oncology, SegResNet

## Abstract

Brain tumor is one of the most challenging neurological diseases to diagnose and even a minor inaccuracy in the tumor characterization can be fatal. An accurate and reliable brain tumor segmentation from 3D MRI images is a fundamental requirement for an effective diagnosis, treatment planning and assessment of outcome in neuro-oncology. Due to infiltrative growth of tumors, heterogeneity in its structure and diffuse boundaries of tumor regions, brain tumor segmentation is quite critical and challenging. Even a minor error in delineation can adversely affect surgical resection and radiotherapy planning. To address these challenges, this study proposes a region-adaptive ensemble framework that integrates the complementary strengths of two capable 3D segmentation models, SegResNet and Swin UNETR through a staged fusion strategy: simple averaging, region-adaptive soft weighting (RSW), and a disagreement-based region-specific refinement (DRE) for high-conflict voxels. The CNN-based SegResNet is capable in capturing fine-grained local textures and well-defined tumor cores due to its convolutional local bias whereas Transformer-based Swin UNETR is capable in modeling long range contextual dependencies across MRI volume due to its hierarchical Transformer architecture. These two models are finetuned on BraTS 2020 dataset and then integrated using a dynamic voxel-wise disagreement-based fusion strategy that adaptively weights model predictions based on parameters like regional confidence, historical performance and level of disagreement. The multi-run experimental evaluation of the architecture on BraTS 2020 dataset is able to achieve impressive dice scores of 0.9447 ± 0.0021 in Whole Tumor (WT), 0.9231 ± 0.0033 in Tumor Core (TC) and 0.9071 ± 0.0041 in Enhancing Tumor (ET) regions. These results indicate that the Region Adaptive fusion with a disagreement-based refinement between convolutional and transformer-based models leads to a robust framework for brain tumor segmentation, that upon further research and validation might turn out to be suitable for clinical decision making and treatment planning.

## 1 Introduction

Brain tumors are one of the most challenging medical crises of our time that often acts like a hidden invader that seamlessly blends into brain’s healthy tissue. Fundamentally, no two tumors look or grow in the exact same way and thus even experts in this field find it difficult to detect and then distinguish boundaries between tumorous regions with naked eye and that too within a limited timeframe. Due to this irregularity, a successful treatment often depends on creating an intelligent deep learning-based technology that could process 3D MRI volume of brain and detect and map exact boundaries of the tumorous sub-regions with high reliability.

There have been researches for over a decade in this field where several deep learning techniques have been developed and implemented for tumor identification and segmentation in MRI images. However, due its irregular shapes and heterogeneity between the intensities of different sub regions, the accurate segmentation of tumor regions is still considered challenging. Even a minor inaccuracy or uncertainty could be catastrophic as incorrect exposure to radiation or any error in surgical removal of tumor tissues may result in long-term neurological deficits or even recurrence of tumor. Thus, there is a pressing need to keep on enhancing accuracy and reliability of the existing architectures while minimizing any room for error. Motivated by this need, we propose a fully automated deep-learning method that leverages disagreement-based adaptive ensembling of CNN (Convolutional Neural Network) and Transformer architectures for detection and accurate segmentation of the various subregions in brain tumor.

The system is basically a disagreement-based adaptive ensemble of two distinct and contrasting deep learning architectures: CNN-based SegResNet and the Transformer-based Swin UNETR. The complementary strengths of SegResNet and Swin UNETR in volumetric segmentation make them an ideal choice for an ensemble. The convolutional inductive bias of SegResNet promotes the extraction of local texture patterns and fine-grained details which is suitable to define the crisp boundaries in compact tumor cores. On the other hand, Swin UNETR processes image patches through a hierarchical encoder-decoder structure that enables it to capture long-range global dependencies and better understand the overall anatomical structure of the brain. Combining these distinct strengths, a robust baseline can be established to handle the extreme structural variability found in the BraTS dataset.

To make the segmentation even more reliable and applicable to real life clinical scenarios, we move beyond a simple averaging of model outputs by employing a region-adaptive ensemble strategy with an additional disagreement-based refinement step for high-conflict boundary voxels. In other words, rather than treating output voxels of every model equally, we calculate outputs for a region by dynamically weighing the contributions of each model based on their confidence and performance in that particular region. By relying on SegResNet for texture-sensitive regions and Swin UNETR for global structural areas, the system ensures that the most appropriate model is applied per region. For cases where significant disagreement exists between the predictions of the individual models, priority is given to the model that is more confident with its prediction. The proposed models are primarily trained and tested using the BraTS 2020 dataset that is later ensembled into a reliable and anatomically consistent CNN–Transformer hybrid framework, which has the potential to improve the accuracy of neuro-oncological interventions, routine radiological diagnosis, and longitudinal monitoring, along with treatment planning.

## 2 Related works

The field of medical imaging including Brain Tumor Segmentation has seen a shift in the paradigm over the past decade with the introduction of the deep learning concepts and CNNs. The early techniques used for identifying and segmenting brain tumors were based on pixel intensity and shape models. These techniques often struggled when tumors had irregular shapes or when they blend into the surrounding healthy tissue. Even though these techniques formed the basis of computer-aided diagnosis, they were not able to address the significant variation in the density and contrast of tumor tissues. Hence, the focus of the research has shifted toward more flexible CNNs and transformer based frameworks that demonstrate the superiority of learned representations over handcrafted features (Havaei et al., 2017; Wulandari et al., 2018; Gurbina et al., 2019; Sun et al., 2019; Choudhury et al., 2020; Arunkumar et al., 2020). Recent studies have explored these frameworks by applying different approaches, including residual learning and self-attention mechanisms to achieve accuracy across the tumor regions. Despite this, it has been difficult to attain precise segmentation due to the blurry nature of the tumor boundaries. This has led to the development of different strategies that combine the models in order to accommodate various brain regions. This has been essential in the development of precise diagnostic tools.

A method for the automated detection of brain tumors was proposed in Reddy et al. (2018). The method utilized a number of image processing techniques. Denoising of MRI scans was performed using median filtering. K-means clustering was used for the segmentation of the brain images. Morphological operators were useful for the effective stripping of the skull. Level sets were also used for the segmentation of the tumors. Although the method was successful in the detection of tumors using intensity changes, it faced difficulties in dealing with tumors of irregular shapes and the presence of tissues around the tumors. The results show that although clustering method is successful, the robustness of the method may not be adequate. Saeedi et al. (2023) and Solanki et al. (2023) have shown substantial improvement in robustness by automatically learning discriminative tumor representations thereby reducing the dependence on handcrafted methods.

Balasamy et al. (2024), Anantharajan et al. (2024) and Fahim et al. (2025) have summarized the rapid evolution of deep learning for brain tumor analysis, covering CNNs, Transformers, hybrid models, explainability and clinical deployment challenges. Fahim et al. (2025), in their research paper, took a deep dive into how the analysis of brain tumors is changing, from simple manual checks to sophisticated 3D segmentation. The authors used a wide range of tools, including conventional CNNs, Transformers, and object detection models like YOLO, to evaluate how effectively these tools handle real-world, messy data from MRI scans. One of the important observations made in this research paper is that most of these deep learning models are “black boxes”. This means it is not easy for a doctor to trust a machine’s diagnosis if he or she does not understand how it arrived at a certain conclusion. This, in itself, is a huge reason why such tools are not being used in hospitals. The authors also observe how a model may give an exceptionally high accuracy on a test dataset but may not perform as well on data from a different hospital, obtained using different imaging devices. The authors’ conclusion is that rather than trying to achieve higher accuracy, researchers need to work toward developing models that are not only robust but also “explainable.” Farhan et al. (2025), in their paper demonstrate XAI-MRI that emphasized on improving the transparency and clinical trustworthiness of automated brain tumor analysis by integrating explainability into ensemble-based segmentation models.

In the research paper presented by Lu et al. (2025), the authors tried to address the issue of brain tumor diagnosis through non-contrast MRI scans. This will, in turn, make the process much easier for patients who are unable to undergo contrast MRI scans. To compensate for the lack of contrast, the authors decided to use the average of the T1 and T2 scans. This provided the system with a much richer set of input details. The authors tried various architectures, and the final one that they decided to use was Darknet53 for the classification of the different types of tumors and ResNet50 for the actual segmentation. This worked remarkably well, achieving a classification accuracy of 98.3% and a mean Dice score of 0.937. This proves that the data fusion technique has the potential to attain high accuracy without the need to undergo invasive procedures. Although the system has not yet been implemented in hospitals, it is a step toward creating a much safer system. Similar observations were also reported by Cuberos and Ochoa Gomez. (2024), who analyzed the impact of different MRI sequences on segmentation performance using BraTS2020.

Instead of relying on the basic 2D representations that are likely to miss the bigger picture, the study in Pal et al. (2024) gave the network a chance to look at the entire slice at once. Through the creation of the SAC-UWNet, using the specialized self-attention block, the researchers enabled the network to consider the relationship between all the pixel positions within the image at the same time, as opposed to looking at the image in small patches. This “global” view is what allows the network to stay “locked onto” the real features of the tumor and ignore the “distracting, low-contrast healthy tissue surrounding the tumor.”

On the other hand, the work in Lu et al. (2023) took a different route to better capture the context. The authors recognized that a brain scan is really just a stack of slices that flow into one another and thus developed M-Net, which treats the slices as if they are continuous. They also developed the “Mesh-Cast” mechanism, which allows information to flow back and forth between the slices. This really helps improve the consistency of the tumor’s shape as it moves through the 3D volume, thus “capturing the temporal-like spatial relationships without the need for the massive computational power required for 3D convolution.”

Unlike previous works, which mainly concentrated on modifying the internal layers of the neural network, the work in Khalil et al. (2020) takes a different route by tackling the “initialization problem.” This problem is essentially about where to start the segmentation curve, which, according to previous methods, would not work unless it is done precisely. This problem was addressed by developing a hybrid model using the dragonfly algorithm with k-means clustering to locate the tumor before the segmentation process begins. This method, unlike previous methods, does not engage in random guessing, as it places the initial contour very close to the real boundaries of the 3D MRI scan. Being bio-inspired, this method does not require large amounts of data, unlike deep learning methods, which are computationally expensive. This optimizes the process, reducing the algorithm’s steps from hundreds to as few as 15.

The research in Pereira et al. (2016) discusses a method of brain tumor segmentation using a CNN that relies on the application of small 3×3 kernels to construct a deeper network without overburdening it with weights. Pereira and his team have tried to address the issue of intensity normalization and data augmentation due to the massive differences in the presentation of gliomas in MRI scans. They have also worked on improving the segmentation results for rare tumor classes by rotating the patches and using data from high-grade tumors to enhance the low-grade tumor models. This approach has worked well for the researchers, who secured first place in the BRATS challenge of 2013 with a 0.88 Dice score for complete tumors. They also secured second place in the BRATS challenge of 2015. The researchers have also observed that applying Leaky ReLU has helped the network learn effectively and refine the segmentation boundaries.

In the research carried out in Purohit and Bhatt (2025), the authors focus on the impact of different deep learning models and optimization techniques on the outcome of brain tumor segmentation. The authors, Purohit and Bhatt, tested various models, such as the U-Net model, the Transformer model, and others, and concluded that the Adam optimizer and Ranger21 optimizer play a crucial role in the training process. By referencing different literature, they concluded that the accuracy of the model, when fine-tuned, can reach as high as 98%, with a mean intersection over union of 0.8665. The authors also concluded that transfer learning can be applied to small datasets, as well as explainable AI, to ensure that the logic behind the model is understandable to medical professionals.

In the study by Cherguif et al. (2019), a novel approach was proposed for the segmentation of brain tumors by creating a modified version of the U-Net architecture through increasing the depth of the convolutional layers from two to three. This was motivated by the segmentation of the whole tumor from the FLAIR images. The performance evaluation of the proposed architecture was performed using the BRATS 2017 dataset, and the experimental results showed that the proposed architecture outperformed the simplified versions of the U-Net architecture by achieving a Dice Similarity Coefficient of 0.81 and an accuracy of 99%. The experimental results showed that the increased depth of the blocks helped in the representation of the tumor boundary.

Moving a step above from individual models, a simple averaging-based ensemble strategy utilizing six distinct 3D models was implemented by Feng et al. (2020). This approach integrated different 3D U-Net architecture and a Dense-Net variant that introduced diversity through varying hyperparameters such as patch size and initial filter count. A voxel wise averaging method was followed to combine the output probability maps of each model aiming to reduce individual model errors and random noise and improving the delineation of tumor sub-regions. Upon evaluating on BraTS 2018 dataset, this ensemble approach was able to achieve Dice Coefficients of 0.926 for whole tumor, 0.911 for the tumor core, and 0.870 for the enhancing tumor. More recently, Javaji et al. (2024) further demonstrated that ensemble learning can improve pediatric brain tumor segmentation by combining complementary model predictions that overcomes individual model biases.

Recent developments have opened doors to focus on overcoming the inherent uncertainties in medical images by incorporating uncertainty quantification into these images. Zhou and Zhu (2023) proposed a multi-encoder 3D U-Net architecture that integrates MC dropout to produce uncertainty maps on medical images. The generated maps can be taken as supplemental structural constraints in refining results from segmenting images. This is an excellent idea as no task-specific thresholds need to be applied, allowing for the adaptive focusing on uncertain areas in images, such as inter-tumor boundaries, where ambiguity in images due to high levels of noise and low contrast leads to misclassification. The studies prove that aleatoric and epistemic uncertainties, when directly accounted for in training, enhance predictions based on both Dice scores and Hausdorff distances.

The DFuse-Net approach was developed further, as disentangled learning of representations for fusion purposes that further advances the paradigm of safe multi-modal fusion methodologies (Zhou et al., 2026). This tool embeds a Disentangled Texture Fusion Module along with a Disentangled Semantic Fusion Module for simultaneously fusing texture and semantic representations of images from different MRI sequences. The approach also involves contrastive-aware learning for the semantic alignment of related representations. Furthermore, it conducts the MC dropout technique for the provision of voxel-wise confidence estimation. This dual-pronged approach of disentangled representations reduces representational redundancy.

Beyond segmentation specific frameworks, recent advancements in medical Artificial Intelligence have also created a scope for robust feature learning and explainable decision-making frameworks. Haq et al. (2023) proposed DCNNBT which is a deep CNN based framework for brain tumor classification that demonstrated the effectiveness of deep feature extraction for neuro-oncological image analysis. More recently, HEAL-LLMF introduced a hierarchical explainable AI framework which combine LLMs with multimodal healthcare information to support personalized clinical decision-making (Haq et al., 2026). These studies demonstrate a growing importance of adding up information sources and improving the interpretability of AI-driven healthcare systems.

Significant progress can be observed in automated brain tumor segmentation moving from the early hand-crafted feature-based methods to the latest advanced deep learning pipelines. Upon surveying existing literature, three recurring limitations are revealed. First, majority of existing research works treat CNN and Transformer based architectures as competing alternatives ignoring their complementary strengths and focus on individual optimization of these architectures, often leading to a diminishing marginal gain in performance. It needs to be considered that each architecture has some unique strengths and weaknesses and neither is inherently superior the other; therefore, they can contribute complementary strengths through different hybrid ensemble techniques. The effectiveness of ensembling CNN and transformer has also been demonstrated in other image analysis tasks, where ensemble learning improved the feature representation by combining complementary local and global information Baskota et al. (2025).

Second, ensemble approaches that do exist such as the multi-model 3D U-Net of M-Net rely exclusively on static averaging of model outputs by implicitly assuming equal confidence of the models across all the voxels and tumor regions. This assumption fails for heterogenous tumor sub-regions such as peritumoral edema and enhanced tumor where static ensembling approaches may often suppress the correct predictions and favour the erroneous ones.

Third, while dynamic uncertainty quantification has been incorporated into segmentation pipelines by Zhou and Zhu via MC Dropout and by DFuse-Net via stochastic confidence estimation, these approaches require multiple stochastic forward passes in inference time that introduces some significant computational overhead. Apart from that, this is applied within single architecture framework and thus lacks a model level diversity in architecture often needed to capture both local and global features simultaneously.

Few advancements can be seen in uncertainty quantification in Zhou and Zhu (2023) and disentangled fusion in Zhou et al. (2026), a critical gap still remains in the balanced integration of global context and local features due to lack of model level diversity in these frameworks. Additionally, they do not explicitly address the severe class imbalance across tumor sub-regions during training that can often bias learning toward dominant regions. There is therefore a need for a hybrid framework that integrates global contextual information with fine-grained characteristics while mitigating the class imbalance through region-overlapping training strategy.

To address these limitations, we propose a dynamic disagreement-based integration strategy where different fusion pathways are followed based on disagreement-based uncertainty between model predictions. In addition to that a region adaptive ensemble strategy is introduced considering the relative capability of individual model across different tumor sub-regions. By integrating complementary strengths of CNN and transformer-based architectures in most effective manner, we propose to develop a proof-of-concept for a segmentation framework that could accurately segment tumor regions in a way that might prove to be applicable to real world clinical scenarios in future.

## 3 Proposed architecture

**Figure 1:**
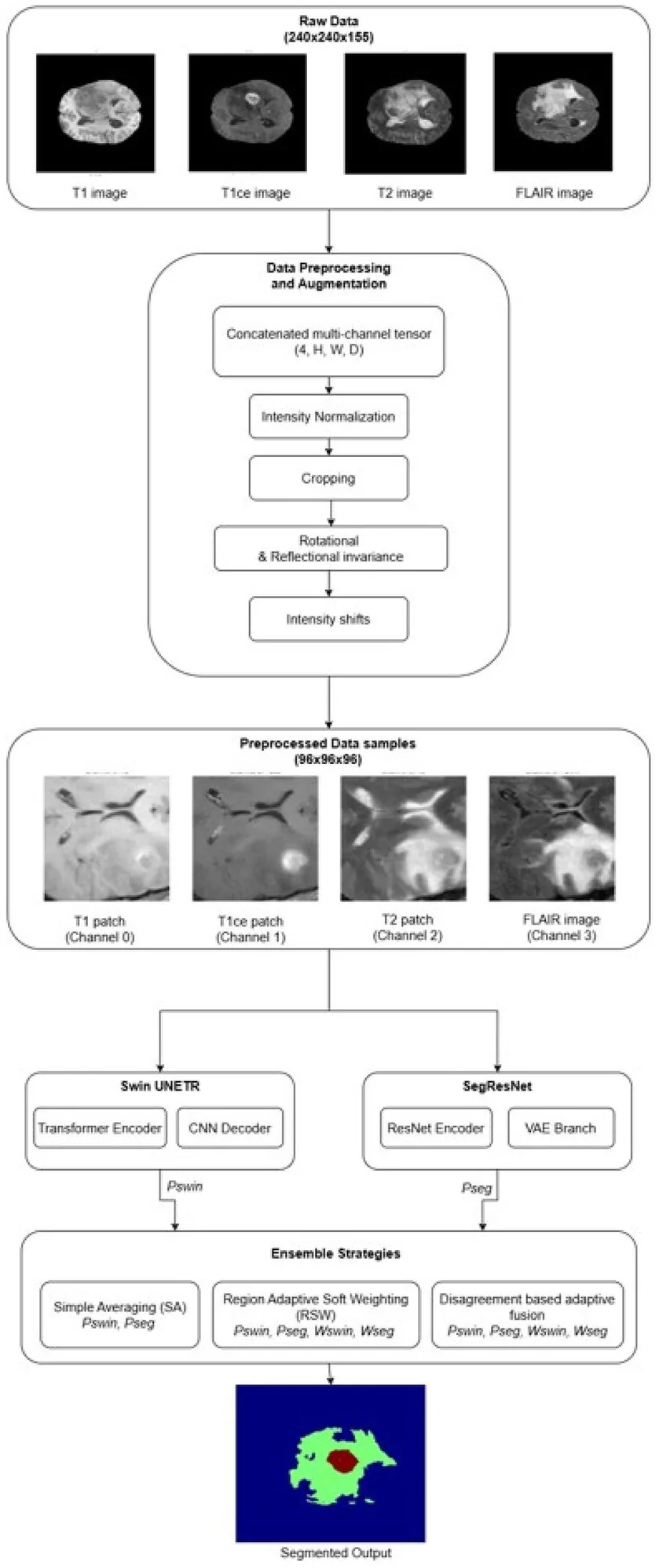
Proposed workflow diagram for brain tumor segmentation.

The architecture proposed in this study for Brain Tumor Segmentation follows a multi-staged pipeline as illustrated in the Figure 1. The four distinct MRI modalities, i.e., T1, T1ce, T2 and FLAIR, originally at a resolution of 240 x 240 x 155 voxels are concatenated into a multi-channel tensor and then pre-processed through series of stages such as intensity normalization and cropping to standard patches of 96 x 96 x 96 voxels. Thereafter, these patches are passed through augmentation phases such as the application of rotational and reflectional invariance and intensity shifts so that model does not overfit to the training data and it could generalize better to real-world noise.

These preprocessed volumetric patches are then fed to the parallel inference engine consisting of two deep learning models: Swin UNETR and SegResNet. Swin UNETR utilizes hierarchical transformer-based encoder coupled with a CNN decoder to capture long range spatial dependencies whereas SegResNet utilizes a ResNet based encoder coupled with additional Variational AutoEncoder (VAE) branch to capture fine-grained local features in the patches. These models independently generate Probability maps P_swin_ and P_seg_ that are utilized by subsequent ensemble stages.

Final segmentation results are obtained by passing intermediate outputs of both the models to either of the three ensemble strategies: Simple Averaging (SA), Region-Adaptive Soft-Weighting (RSW) and Disagreement-based Region-Specific Fusion (DRE). Finally comparative analysis is done to find out which ensemble strategy can best utilize strengths and overcome weaknesses of individual models to give the best segmented output. Overall, the primary contribution of this framework is the Region-Adaptive Soft-Weighting Ensemble technique and the additional disagreement based fusion step in DRE is aimed to provide an incremental refinement to this technique in high-conflict boundary regions.

## 4 Methodology

The methodology of this study involves a systematic pipeline that was followed to develop the entire architecture to accurately segment tumor regions from 3D MRI volumes. This pipeline involves the selection and analysis of the BraTS 2020 dataset followed by preprocessing and augmentation of the data samples to get inputs in desired format for the models. The preprocessed data patches were used to train two complementary deep-learning architectures: CNN based SegResNet and transformer based Swin UNETR. The pretrained weights training hyperparameters were adjusted unique to each model considering their nature. These finetuned models were ensembled in three different ways: simple averaging, region specific weighting and disagreement-based adaptive ensembling. Finally, all the models and ensemble architectures were tested in unseen BraTS dataset using a suite of performance metrics.

### 4.1 Dataset selection and analysis

In this study the BraTS 2020 (Brain Tumor Segmentation) dataset was selected from Kaggle (2020) as it consists of clinically acquired MRI scans from multiple institutions that would ideal for the training and evaluation of state-of-the-art segmentation architectures. While more recent versions of BraTS datasets have been released, the 2020 iteration was specifically chosen as it remains as a foundational benchmark for 3D segmentation tasks. Unlike the later version that focus on niche sub-challenges such as image synthesis, missing modality completion, or underrepresented populations, the 2020 dataset acts as a standard baseline for general segmentation of tumor regions. Additionally, BraTS 2020 dataset has been extensively utilized in multiple researches allowing us to make an extensive comparison against the established state-of-the-art architectures.

**Figure 2:**
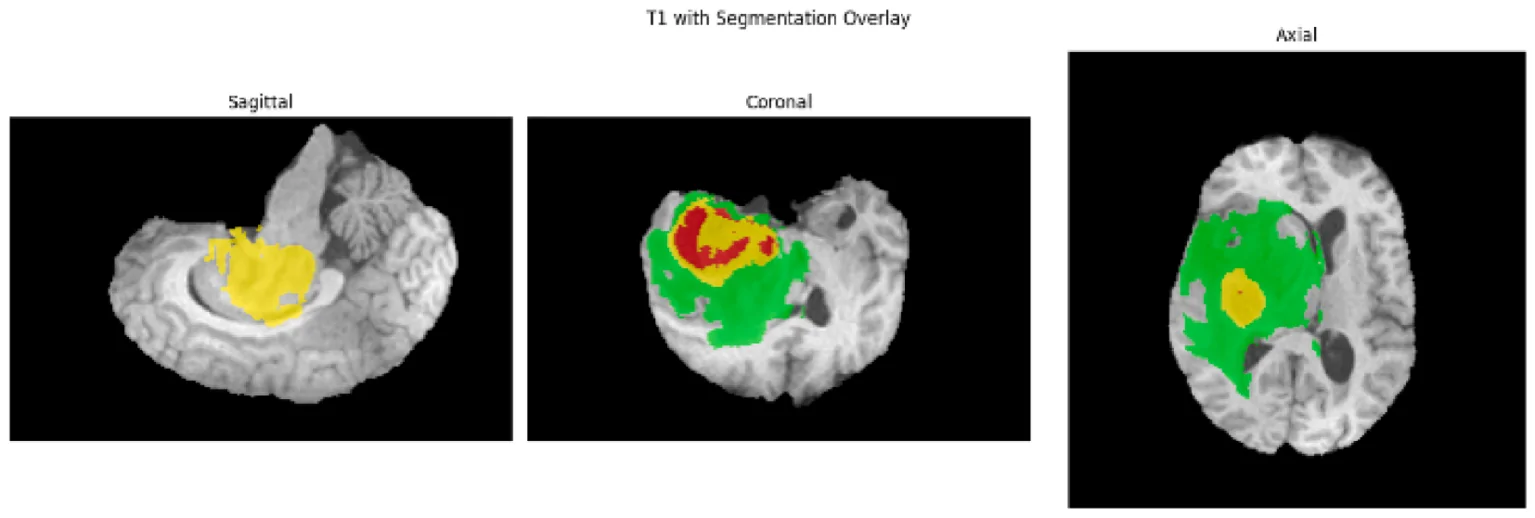
3D views of T1 volume with segmentation overlay (green = WT, yellow = TC, red = ET).

**Figure 3:**
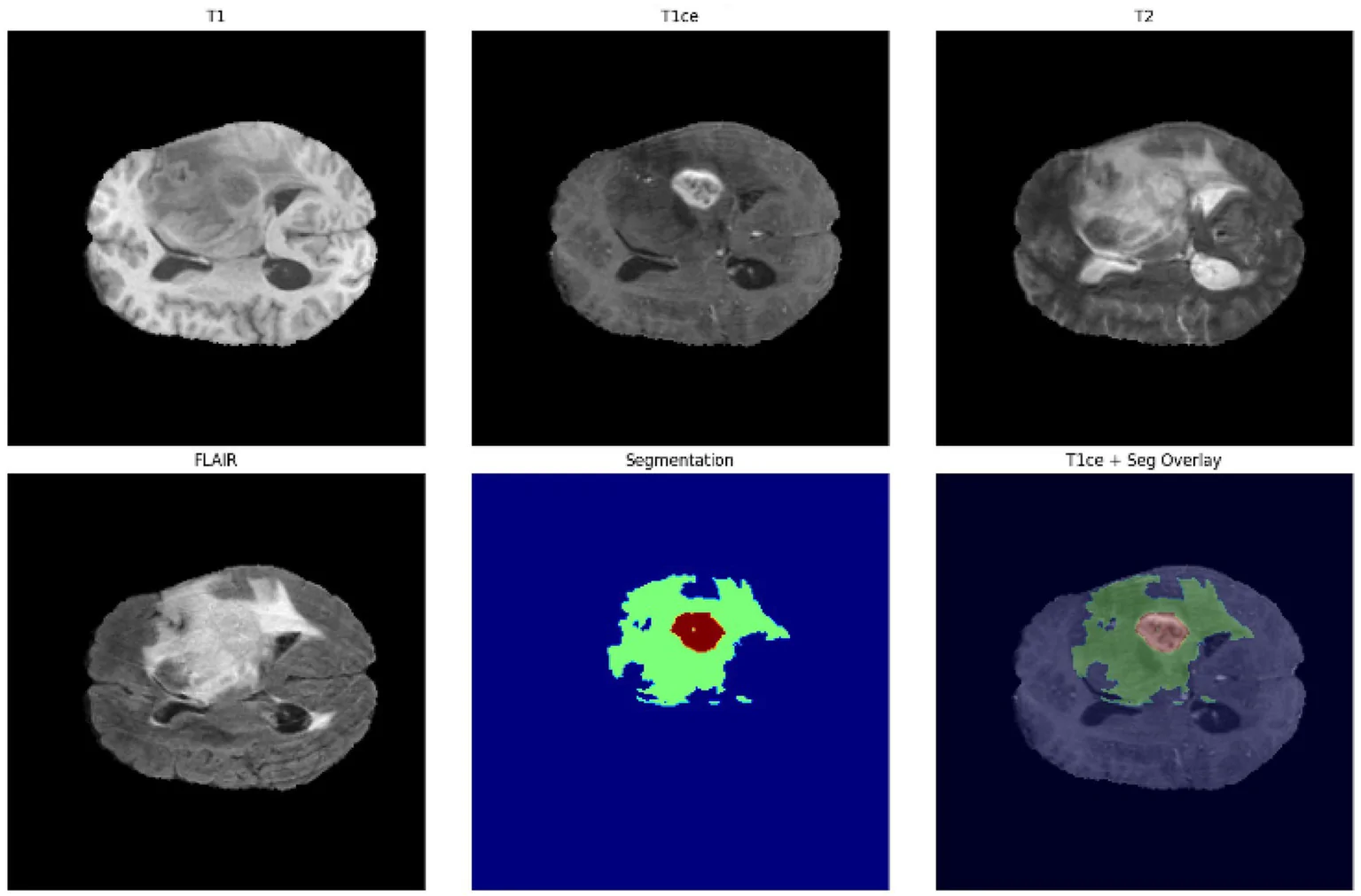
Sample data modalities and segmentation map (green = WT, red = TC, yellow = ET).

A subset of 368 valid patient samples were taken from BraTS 2020 where each patient case consisted of four 3D MRI modalities: Native (T1), post-contrast T1-weighted (T1ce), T2-weighted (T2) and Fluid Attenuated Inversion Recovery (FLAIR). Figure 2 demonstrates the 3D views of T1 volume through three different angles: Sagittal, Coronal and Axial. Figure 3 demonstrates a sample patient data that includes the four different modalities, segmentation map for the tumor regions and an overlay of T1ce and segmentation map.

**Figure 4:**
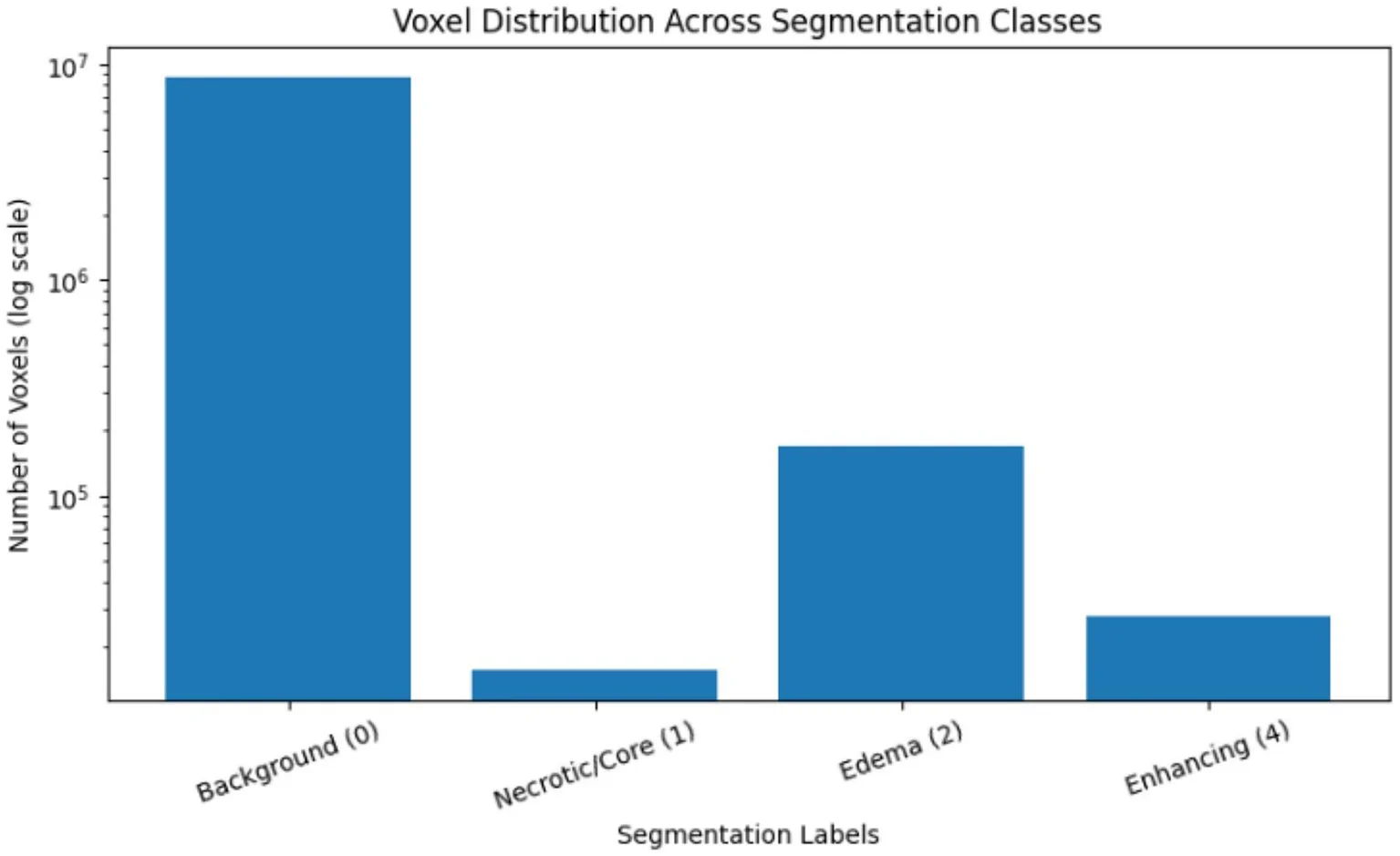
Voxel distribution across segmentation classes.

The 3D segmentation map has 4 different labels denoting background and 3 different tumor regions. The voxel distribution across these labels has a severe class imbalance with 8,716,021 voxels for background, 15,443 voxels for Necrotic/Core, 168,794 voxels for Edema and 27,742 voxels for Enhancing region of tumor for a sample data. The background class dominates voxel distribution, while tumor related classes occupy significantly lesser number of voxels which is typical in brain tumor segmentation tasks. Figure 4 is a bar graph with logarithmic scale to effectively visualize this severe class imbalance. This imbalance requires cropping out of excess background voxels to let model focus on the tumor regions more.

**Figure 5:**
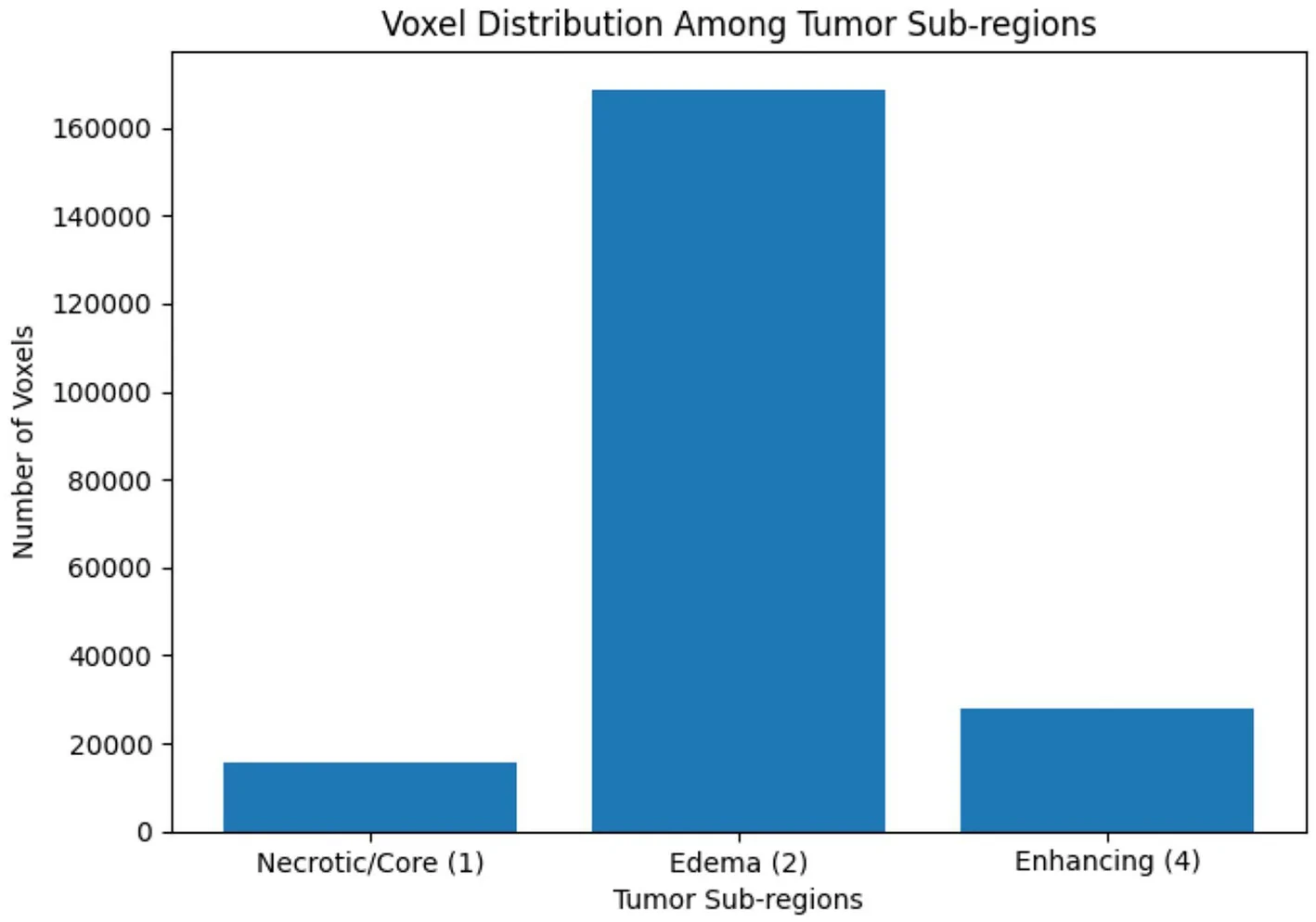
Voxel distribution among tumor sub-regions.

To further analyze the imbalance within the tumor regions, Figure 5 demonstrates the voxel distribution restricted to just the tumor labels. We can see that even within the tumor region, edema constitutes the largest portion among all tumor classes followed by enhancing tumor and necrotic/core region. This intra class imbalance further emphasizes the need of overlapping region-based training and evaluation to reduce any bias in model training.

**Figure 6:**
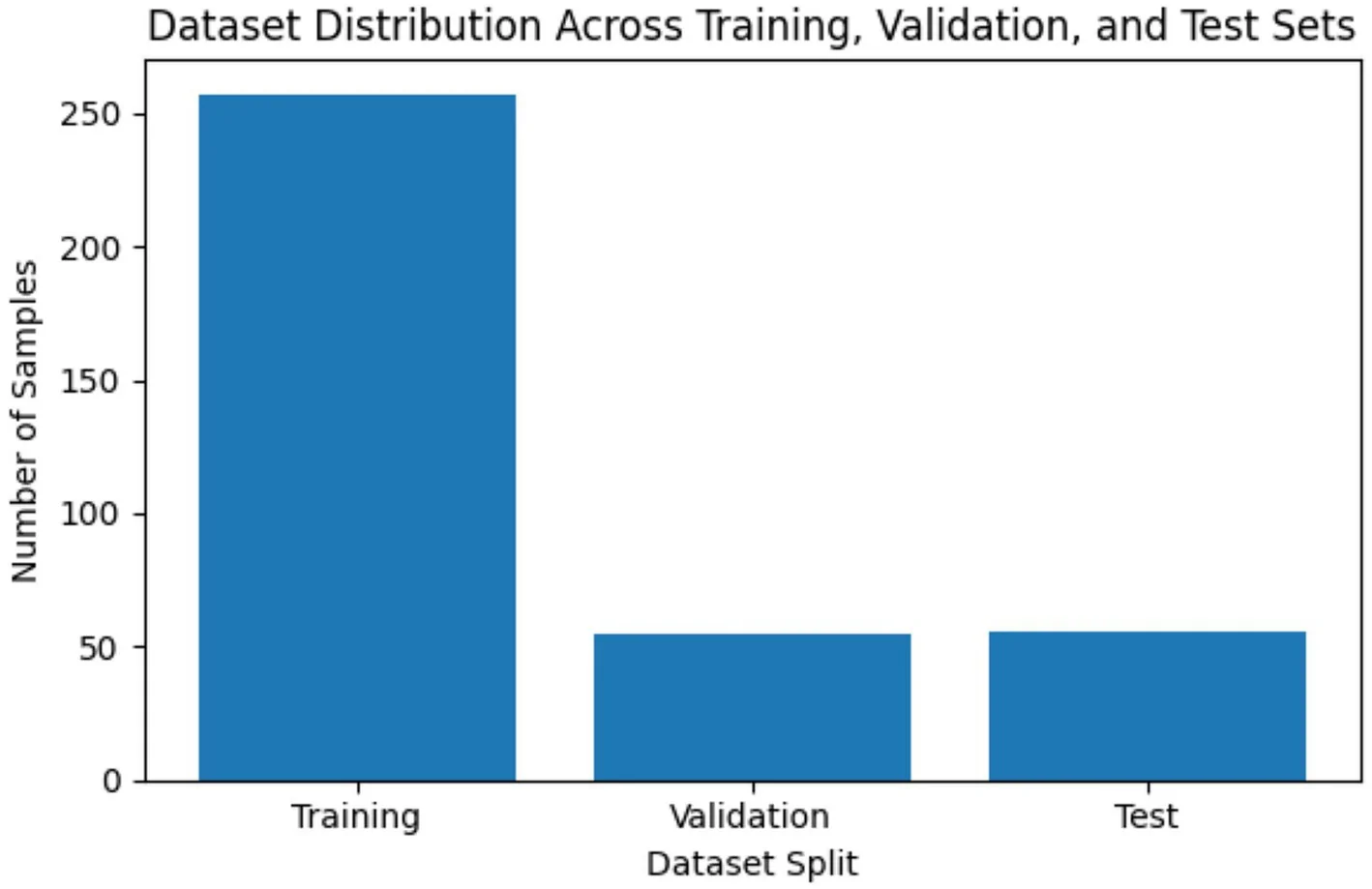
Dataset distribution across train, validation, and test sets.

All volumes were provided with a resolution of 240×240×155 voxels and a standardized voxel spacing of 1.0×1.0×1.0 mm^3. The dataset was partitioned into 257 Training, 55 Validation, and 56 Testing sets as indicated in the bar plot in Figure 6.

### 4.2 Data pre-processing

To ensure the deep learning models effectively extract features across different sequences, a rigorous pre-processing pipeline was implemented using the MONAI (Medical Open Network for AI) framework.

#### 4.2.1 Modal concatenation and label transformation

The four MRI modalities (T1, T1ce, T2, FLAIR) were concatenated into a single multi-channel tensor of shape (4, H, W, D) which would allow the models to learn cross-modal correlations simultaneously.

The original segmentation masks provided in BraTS 2020 contained four discrete labels: 0 (Background), 1 (Necrotic/Non-enhancing Tumor Core), 2 (Peritumoral Edema), and 4 (Gd-enhancing Tumor). These labels were transformed into three overlapping regions Whole Tumor (WT), Tumor Core (TC), and Enhancing Tumor (ET). WT was taken as combination of all tumor labels (1, 2 and 4), TC was taken as combination of labels 1 and 4, whereas ET was taken as label 4 exclusively. This label transformation into overlapping tumor sub-regions helps mitigate model bias due to heavy class imbalance between tumor patches.

#### 4.2.2 Spatial normalization and intensity scaling

To account for varying scan orientations, all volumes were resampled to an isotropic resolution of 1.0 mm^3 and re-oriented to the RAS (Right, Anterior, Superior) coordinate system. To reduce computational overhead and eliminate uninformative background voxels, we applied a foreground-based cropping technique centered on the brain mass.

Raw MRI signals often lack a standardized unit of measure. As the dataset was from multiple institutions, there could be a significant intensity drift across these different scanners. To address this issue, we normalized the intensity values using Channel-wise Z-score Normalization (zero mean and unit variance). This normalization was applied on a per-channel basis specifically ignoring the non-brain background (0 intensity) voxels. This ensured that the statistical distribution of tumorous tissue was not skewed by the background which constitutes the majority of the 240x240x155 volume.

For each modality m ∈ {T1, T1ce, T2, FLAIR}, the voxel intensities (Equation 1) were transformed as:


$$I_{m}^{\prime }=\frac{I_{m}-\mu_{m,\text{nonzero}}}{\sigma_{\left\{m,\text{nonzero}\right\}}}\;$$ (1)

#### 4.2.3 Stochastic augmentation and patch sampling

To mitigate overfitting on this relatively smaller training set and simulate the variability of clinical scans, we applied a suite of stochastic transforms. First, we extracted patches of size 96 x 96 x 96 maintaining a 1:1 sampling ratio between patches centered on tumorous voxels and the background voxels. This ensured that the models receive sufficient signal from the highly imbalanced minority classes (ET and TC). Next, rotational and reflectional invariance was enforced by subjecting the patches to random axial flips and 90degree rotations across the X, Y, and Z planes with a probability of *p* = 0.5. Finally, we applied random shifts in voxel intensities with a scaling factor of 0.1, introducing approximately pm10 shifts in voxel intensity. This simulates the hardware-induced signal-to-noise variations that is common in routine clinical workflows.

**Figure 7:**
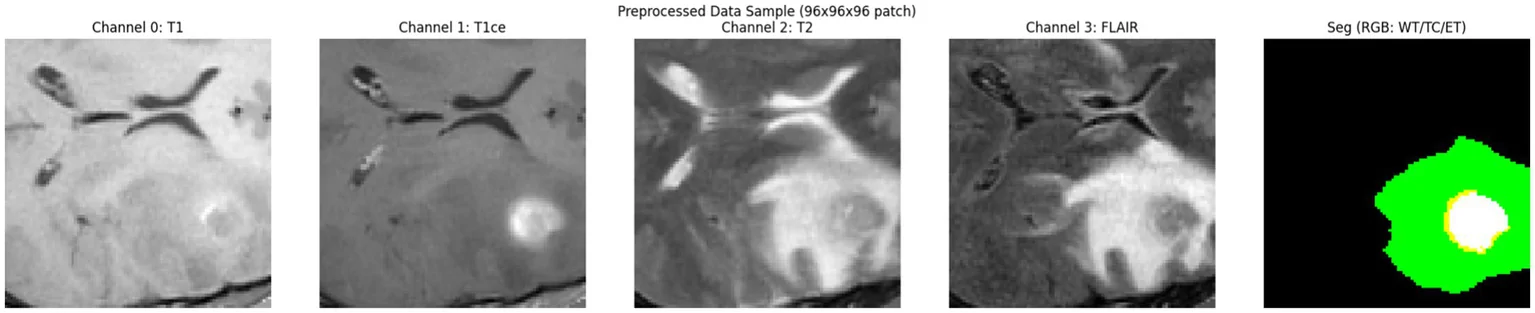
Preprocessed data sample.

Figure 7 consists of a preprocessed patch of MRI volume of the dimension 96 x 96 x 96. We can notice that majority of unnecessary background voxels are cropped out keeping the tumorous region at centre. In addition to that minor augmentaton and artificially introduced noise can be seen in the patch to ensure model does not overfit in the training data.

### 4.3 Model architecture and training

This study implements a hybrid approach that is based on two distinct deep learning architectures to handle different aspects of 3D brain tumor segmentation:

#### 4.3.1 SegResNet

SegResNet is a CNN based encoder-decoder architecture that uses ResNet blocks to maintain strong gradient flow and group normalization to handle the small batch sizes inherent in 3D data. As a result, it is well suited at capturing local spatial hierarchies and fine-grained boundary details in volumetric image which are important for delineating tumor sub-regions.

#### 4.3.2 Swin UNETR

It is a hierarchical Swin Transformer that treats the 3D volume as sequence of patches and is capable of capturing global context and long-range dependencies. This makes it very effective to at understanding the overall anatomical structure and identifying diffused boundaries such as the “Peritumoral Edema”.

#### 4.3.3 Multistage finetuning

Both the models were trained using a multistage training approach that involved a short warmup where encoder layer was frozen and learning rate was linearly increased to stabilize the decoder head followed by full model training with cosine annealing.

##### 4.3.3.1 SegResNet training

In order to stabilize training on smaller dataset and leverage the prior anatomical knowledge for fine grained classification, we initialize this model using weights pre-trained on the BraTS 2018 dataset. Warmup was done for first 10 epochs by freezing the encoder weights. Afterward, the entire network is unfrozen to fine-tune the weights for the overlapping tumor sub-regions (WT, TC, ET). The overall training was done for 65 epochs and the model achieved best mean validation dice of 0.8850 at 56th epoch. During training VAE reconstruction branch was used as an auxiliary regularizer so that encoder could learn more structural latent representations. This was later disabled in inference time with only segmentation decoder used to produce final predictions.

##### 4.3.3.2 Swin UNETR training

Swin UNETR was initialized with pretrained weights from BTCV dataset that would provide a generic anatomical knowledge but still support learning and adaptation to the global contextual relationship within BraTS2020 dataset. Since CT scans are single-channel and MRIs are four-channel (T1, T1ce, T2, and FLAIR), we duplicated the single-channel weights across all four MRI input channels and normalized the scale by ¼ to preserve the backbone’s feature extraction capability. Layers with incompatible dimensions, including the BTCV segmentation head, were reinitialized and trained during fine-tuning. Warmup was done for the first 20 epochs by freezing the encoder weights with linear increase in learning rate followed by full model training. To preserve the generic features learned in the Transformer, we freeze the early encoder stages (Stage 0 and 1) and the patch embedding layer for entire training process. Although BTCV pre-training is based on CT, we keep the early encoder stages and patch embedding frozen to preserve generic features such as edges and organ boundaries detection, while allowing deeper layers and the decoder to adapt to MRI appearance leading to some marginal gains in performance. The overall training was done for 80 epochs and the model achieved best mean validation dice of 0.9120 at 64th epoch.

The pretrained weights were taken from BraTS 2018 for SegResNet so that it could benefit from prior knowledge and better capture fine grained tumor details capture whereas more generic BCTV weights were used for Swin UNETR allowing it to learn global contextual dependencies in the dataset from scratch. As a result, performance differences between SegResNet and Swin UNETR would be jointly influenced by both architectural inductive biases and the distinct pretraining methodologies. Therefore, results should not be interpreted as a pure comparison of CNNs versus Transformers, but as a comparison between two practically tuned, pre-trained backbone architectures.

Training was performed using Mixed Precision to maximize GPU memory efficiency whereas validation was done using a Sliding Window Inference protocol with an ROI of 96x96x96 and a 50% overlap. This would ensure that the model processes the entire 3D volume while maintaining the local context it learned during training.

The epoch wise training loss, validation loss and mean dice was recorded throughout the training process for both models. These records were then mapped into Train loss vs. Validation loss curve and Validation Dice curve for each model.

### 4.4 Metrics and evaluation

The model training and evaluation was done with an optimized set of hyperparameters and performance metrics. The loss function and optimization metrics along with performance evaluation metrics are discussed below.

#### 4.4.1 Loss function and metrics

We utilize a composite Dice-Cross Entropy (DiceCE) Loss that combines the voxel-wise precision of Cross-Entropy with the Dice coefficient’s ability to handle the severe class imbalance between the tumor region and healthy region.

The total loss L_total_ is the sum of the regional Dice loss and the voxel-wise Binary Cross-Entropy (BCE) loss (Equation 2):


$$L_{\text{total}}=L_{\text{Dice}}+L_{\mathrm{BCE}}$$ (2)

The dice component for a predicted volume P and ground truth G is defined as (Equation 3):


$$L_{\text{Dice}}=1-\frac{2\Sigma_{i=1}^{N}P_{i}G_{i}+\varepsilon }{\Sigma_{i=1}^{N}P_{i}^{2}+\Sigma_{i=1}^{N}G_{i}^{2}+\varepsilon }$$ (3)

Where $\in =10^{-5}$ is a smoothing factor to prevent division by zero.

We use Sigmoid activation for the final layer because our target regions (Whole Tumor, Tumor Core, and Enhancing Tumor) are overlapping and a sigmoid allows each voxel to be evaluated for each region independently.

#### 4.4.2 Optimization and learning rate

Initially during warmup stage for each model, a Sequential Learning Rate strategy was applied. The linear warmup was followed by cosine annealing decay to refine the weights as the training converges.

Both models were trained using the AdamW optimizer with a weight decay of 10^−5^ for better regularization. To preserve pre-trained features while allowing the decoder to adapt, a differential learning strategy was adapted where lower learning rate was used for the encoder compared to decoder.

The weight update for a parameter *θ* at step t follows (Equation 4):


$$\theta_{t}=\theta_{\left\{t-1\right\}}-\eta \cdot \nabla L(\theta_{\left\{t-1\right\}})$$ (4)

Where L is the loss function and the gradient $\nabla L(\theta_{\left\{t-1\right\}})$ gives the direction toward which the loss reduces.

*η* is learning rate. For Swin UNETR, η = 5*10^−5^ is taken for encoder layers and η = 10^−4^ is taken for decoder layers. For SegResNet, η = 2*10^−5^ is taken for down sampling path and η = 5*10^−5^ is taken for up sampling path.

For Swin UNETR, we additionally applied Stochastic Weight Averaging (SWA) during the final 5 epochs to improve generalization by maintaining an exponential moving average of model weights with a fixed low learning rate, and used the averaged weights for validation and testing.

#### 4.4.3 Performance evaluation metrics

To measure the performance of the individual models and the proposed ensemble frameworks, we utilize a comprehensive suite of metrics. These metrics evaluate both the overlap accuracy and the reliability of the segmentation boundaries across the three target sub-regions (WT, TC, ET).

We define P as the predicted binary mask and G as the ground-truth manual segmentation. The evaluation is based on the following four cardinal values: True Positive (TP), True Negative (TN), False Positive (FP), and False Negative (FN).

As Overlap and Similarity metrics we use Dice Similarity Coefficient (DSC) and Intersection over Union (IoU).

Dice Similarity Coefficient (DSC) (Equation 5) is the primary evaluation metrics used in this study. It measures the spatial overlap between prediction and ground truth. It is considered most appropriate metric for this study as it provides a balanced assessment for small and irregular tumor structures by equally penalizing false positives and false negatives.


$$\mathrm{DSC}=\frac{2|P\cap G|}{|P|+|G|}=\frac{2\mathrm{TP}}{2\mathrm{TP}+\mathrm{FP}+\mathrm{FN}}$$ (5)

Intersection over Union (IoU) (Equation 6) measures the ratio of the intersection to the union of the two sets.


$$\mathrm{IoU}=\frac{|P\cap G|}{|P\cup G|}=\frac{\mathrm{TP}}{\mathrm{TP}+\mathrm{FP}+\mathrm{FN}}$$ (6)

While IoU is also a useful overlap metric, it could be sensitive to small mismatches and often provides lower scores for small tumor regions.

As Voxel-wise Classification metrics we use Precision, Sensitivity and Pixel-wise Accuracy.

Precision (Equation 7) quantifies the model’s ability to avoid “over-segmentation” by measuring the proportion of predicted tumor voxels that are actually part of the tumor. High precision indicates low false positives, which ensures that minimal clinical intervention is required.


$$\text{Precision}=\frac{\mathrm{TP}}{\mathrm{TP}+\mathrm{FP}}$$ (7)

Sensitivity (Equation 8) measures the model’s ability to identify all actual tumor voxels, which is critical for ensuring no cancerous tissue is missed during surgical planning.


$$\text{Sensitivity}=\frac{\mathrm{TP}}{\mathrm{TP}+\mathrm{FN}}$$ (8)

Pixel-wise Accuracy (Equation 9) represents the ratio of correctly predicted voxels to the total number of voxels in the 3D volume.


$$\text{Accuracy}=\frac{\mathrm{TP}+\mathrm{TN}}{\mathrm{TP}+\mathrm{TN}+\mathrm{FP}+\mathrm{FN}}$$ (9)

However, as MRI volumes are dominated by background voxels, accuracy could be misleadingly high even in case of poor segmentation results. Thus, accuracy is not considered as primary performance indicator in this study.

All these metrics were used to evaluate the performance of the models as well as ensemble frameworks in each of tumor sub regions, i.e., WT, TC and ET.

### 4.5 Ensemble framework

To maximize the predictive performance and clinical reliability of the system, we move beyond individual model outputs by implementing a multi-staged ensemble strategy.

As a baseline, we first implemented three standard ensembling methods. These techniques combine the output probability maps of individual models, where *P_Seg_(v, c)* is the probability assigned by SegResNet to a voxel *v* for class *c*, and *P_Swin_(v, c)* is the corresponding probability from Swin UNETR.

#### 4.5.1 Simple averaging (SA)

Here both models were treated equally capable, and the final ensemble probability P_SA_ (Equation 10) is calculated as the arithmetic mean of individual probabilities.


$$P_{\mathrm{SA}}(v,c)=\frac{P_{\mathrm{Seg}}(v,c)+P_{\text{Swin}}(v,c)}{2}$$ (10)

While this reduces random noise, it might fail to account for the specific anatomical strengths of each model.

#### 4.5.2 Region-specific weighted ensemble (RSW)

In this method, we assign fixed normalized weights *ω* (Equation 11) based on the validation performance of the model *m ∈ {Swin, Seg}* for each tumor sub-region *c ∈ {WT, TC, ET}* as:


$$w_{\left\{m,c\right\}}=\frac{D_{\left\{m,c\right\}}}{D_{\left\{\text{Swin},c\right\}}+D_{\left\{\mathrm{Seg},c\right\}}}$$ (11)

Then we compute the ensemble probability (Equation 12) map for each voxel *v* as:


$$P_{\mathrm{RSW}}(v,c)=\omega_{\mathrm{Seg}}\cdot P_{\mathrm{Seg}}(v,c)+\omega_{\text{Swin}}\cdot P_{\text{Swin}}(v,c)$$ (12)

Here, $\omega_{\mathrm{Seg}}+\omega_{\text{Swin}}=1$ .

This ensures that the architecture with a higher historical accuracy for a specific core or edema region has a proportionally higher influence in the resulting probability score.

#### 4.5.3 Disagreement-based region-specific ensemble (DRE)

This hybrid framework integrates region-specific expertise of a model with a dynamic disagreement-based heuristic uncertainty handling mechanism, i.e., disagreement (Equation 13) is calculated as level of disagreement between models, i.e., the absolute difference between the Segresnet and Swin UNETR outputs for voxel *v* region *c*.


$$d(v,c)=|P_{\mathrm{Seg}}(v,c)-P_{\text{Swin}}(v,c)|$$ (13)

High values of d typically highlight tumor boundaries or low-contrast regions where the local texture (CNN) and global context (Transformer) conflict.

If the disagreement is less than a threshold, weighted fusion is done where the system utilizes regional weights (*ω*) combining SegResNet’ s boundary precision with Swin UNETR’s structural coherence. But in areas of high uncertainty, the ensemble selects the model output with higher confidence (furthest from the 0.5 decision boundary). We acknowledge that in rare cases where both models assign probabilities close to 0.5 (low confidence), slightly confident model is chosen arbitrarily. Such cases occur in highly ambiguous regions and can be minimized by involving more diverse models in the hybrid architecture and even warranting human review in case of clinical settings.


$$P_{\mathrm{DRE}}(v,c)=\{\begin{array}{c} \sqrt{\underset{\text{Weighted Fusion}}{\underset{︸}{\omega_{\mathrm{Seg}}\cdot P_{\mathrm{Seg}}(v,c)+\omega_{\text{Swin}}\cdot P_{\text{Swin}}(v,c)}}\;\text{if}\;d(v,c)<\tau }\\ \underset{\text{Confidence Selection}}{\underset{︸}{{\text{argmax}}_{\left\{P_{\mathrm{Seg}},P_{\text{Swin}}\right\}}(|P-0.5|)}}\;\text{if}\;d(v,c)\geq \tau \end{array}$$ (14)

where, argmax(|P-0.5|) denotes prediction of model with higher confidence in prediction

and *τ* is the disagreement threshold.

To provide more clarity, Algorithm 1 summarizes the voxel-wise disagreement-based fusion procedure that combines SegResNet and Swin UNETR predictions into final segmentation mask.

Algorithm 1: Proposed fusion strategy for disagreement based region adaptive ensemble.
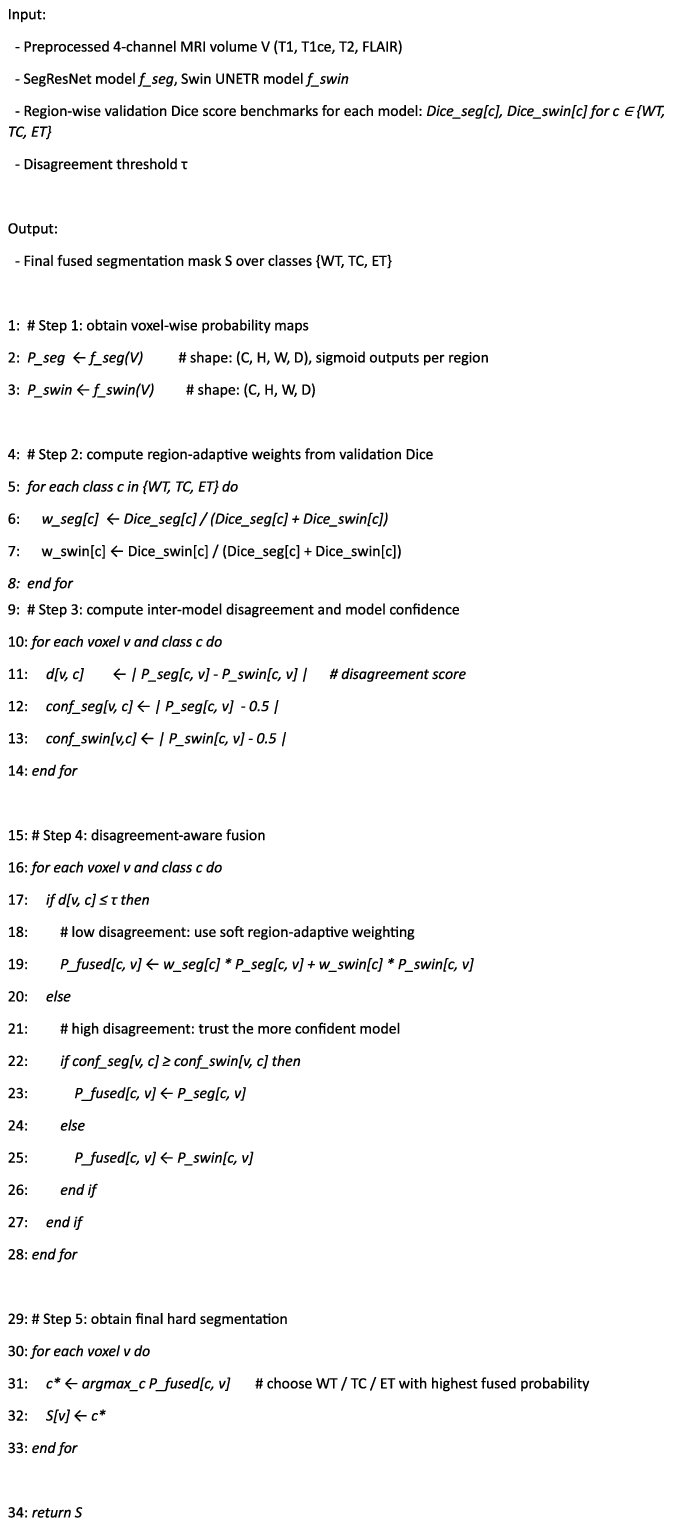

To better analyze the reproducibility and variance, the models were trained, tested and ensemble architectures were evaluated independently across three independent runs using different random seeds for patch sampling and stochastic augmentation. The region adaptive weights for a run were calculated from validation dice score of individual models in that particular run. The threshold 𝜏 for each run was set based on sensitivity analysis of mean dice scores across 0.1 < 𝜏 < 0.9. Since the final layer of both models utilizes a sigmoid activation function, a disagreement value of | P_Seg_(v, c) - P_Swin_(v, c) | > = 𝜏 would signify a fundamental binary conflict where a more confident model needs to be given priority. No test set data were used at any stage of model selection or hyperparameter tuning and test evaluation was done only once after all parameters were fixed.

Likewise, we evaluated multiple methods of ensembling Swin UNETR and SegResNet models across all tumor sub regions and a comparative analysis was done between the ensemble strategies. After metrics-based evaluation, actual segmentation outputs were recorded for both models and ensemble frameworks for visualization and comparison purposes.

## 5 Results and discussion

### 5.1 Training dynamics

The entire training progress for both the models was monitored via loss curves for training and validation along with validation dice scores so that model’s stability and effectiveness of training strategy can be evaluated.

Furthermore, for reproducibility of the proposed ensemble framework and to rule out the possibility that the random initialization was favorable, all three ensemble strategies were evaluated across three independent experimental runs where in each run the individual models were retrained from a common pretrained weights and common set of hyperparameters but using different random seeds for patch sampling and stochastic augmentation. The ensemble weights and disagreement threshold were calculated again based on the validation score achieved in that particular run.

**Figure 8:**
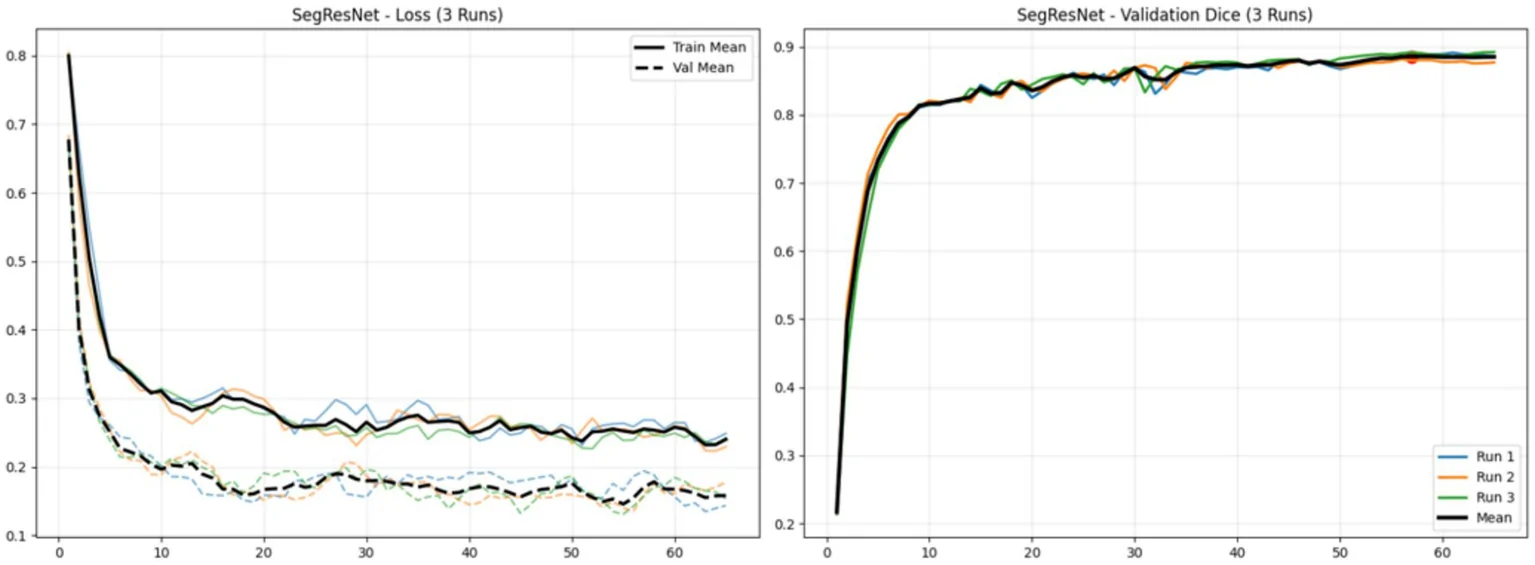
Individual and mean loss curves and validation Dice curve for SegResNet across three training runs.

As we observe in Figure 8, over the 65 epochs training duration across all 3 training runs of SegResNet model, a rapid initial descent was observed followed by a stable plateau of loss curves. As a result of encoder freezing, linear scheduling of learning rate during the initial 10 epochs, the pre-trained weights were preserved and a smooth rise in the dice score was observed. The BraTS 2018 initialization provided a significant head start allowing model to bypass the standard exploration phase needed in a random initialization and leading it to quicker plateau. The validation Dice curve had a smooth, logarithmic growth and best mean validation Dice score of 0.8825 was achieved at epoch 57 upon averaging the metrics of all 3 runs. Throughout the learning, a narrow gap is maintained between the training and validation loss curves that indicates that the model was able to learn generalizable features of 3D MRI volumes rather than overfitting to the training sample.

**Figure 9:**
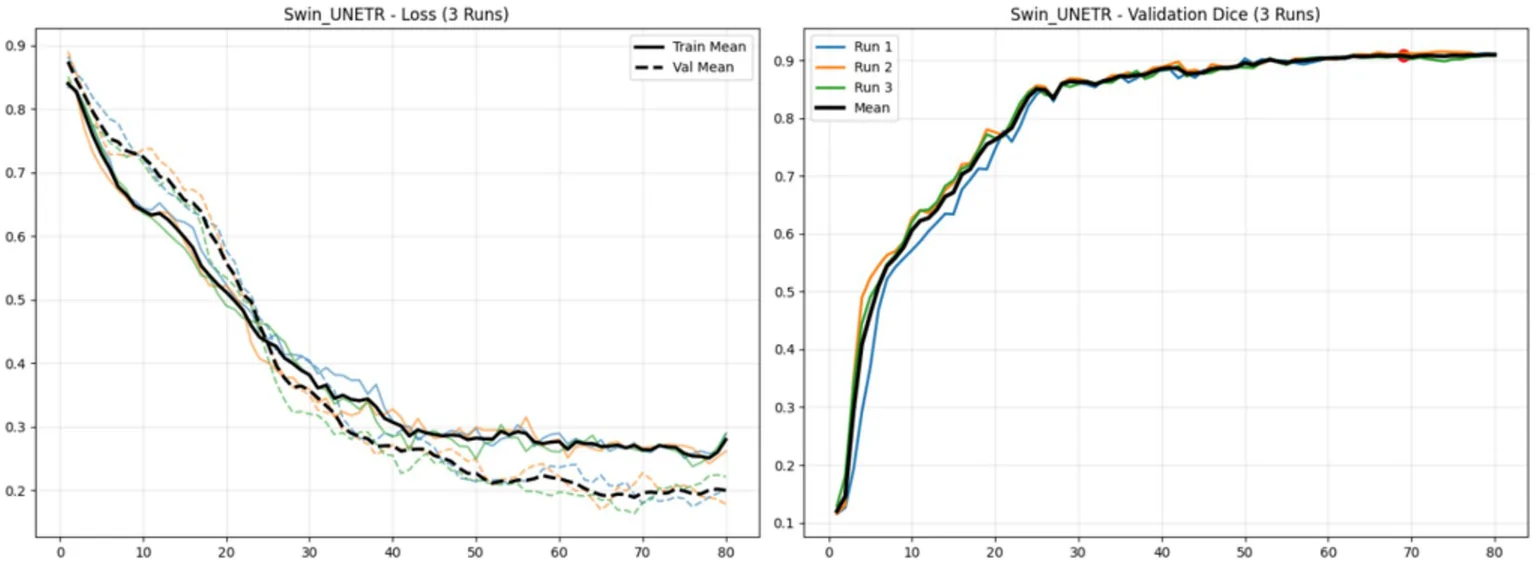
Individual and mean loss curves and validation Dice curve for Swin UNETR across three training runs.

Figure 9 represents the training dynamics for Swin UNETR model across 3 independent training runs. The Swin UNETR was trained for a longer duration (80 epochs) using pre-trained BCTV weights allowing a more gradual learning of global contextual features from the dataset. Compared to SegResNet, it took a longer time to reach peak performance, likely due to the use of lesser task-specific pre-training than the BraTS2018 weights used for SegResNet. This also typical for transformer-based models as they learn global structural relationships through self-attention mechanisms rather than having a local convolutional bias. The initial warmup of 20 epochs with Linear Scheduling of learning rate resulted in smooth rise of dice score while preserving the sensitive encoder weights. The model was able to achieve peak validation Dice score of 0.9113 at epoch 68. Also, the key observation is the stabilization of Dice score on the final epochs (75–80) as a result of Stochastic Weight Averaging (SWA) at a constant low learning rate that helped it achieve a flatter local maximum in dice score.

Across the three independent runs, both the models show a consistent convergence in the final epochs. In the intermediate epochs, Swin UNETR demonstrates a slightly higher variance in the loss and dice values compared to SegResNet, indicating that transformer architectures are more sensitive during fine-tuning. Also, this could be also because the pretrained weights of Swin UNETR (from BTCV) need more adaptation to the BraTS domain leading to minor variance in the loss paths.

Overall, Segresnet demonstrates a high efficiency rapid convergence making it texture expert for compact tumor regions whereas Swin UNETR aided by SWA achieves gradual but higher peak validation score suggesting its global contextual learning over a sufficient training time. It is worth noting that the validation loss plateaus above zero that can be due to the irreducible noise and the intrinsic difficulty of precisely segmenting small ET regions. In such settings, zero validation loss is neither expected nor necessary for a strong Dice performance.

### 5.2 Baseline model performance analysis

The quantitative evaluation of Swin UNETR and SegResNet across the three primary tumor sub-regions: Whole Tumor (WT), Tumor Core (TC), and Enhancing Tumor (ET) displays some distinct performance characteristics for each of these architectures. These differences in capabilities could be jointly influenced by the architectural differences between the models, the difference in the pre-trained weights as well as the difference in fine-tuning pipeline for the backbone models. These differences should not be interpreted as a pure comparison between the individual architectures because the backbone models differ not only in architecture but also in the pre-training weights used during the finetuning process.

Table 1 above provides the mean evaluation result of the individual models along with standard deviation across three independent runs. Both the models achieve a competitive dice score with minimal variation. Swin UNETR demonstrates superior performance in the WT region, achieving a mean Dice score of 0.9351 and an IoU of 0.8774, noticeably outperforming SegResNet (Dice: 0.9125). This suggests that the Swin UNETR backbone in this study is highly effective at delineating the overall tumor extent, which typically covers a larger and more continuous area.

**Table 1:** PER region metrics for SegResNet and Swin UNETR (mean ± SD over three independent runs).

Tumor region	Model	Dice Score	IoU Score	Accuracy	Precision	Sensitivity
Whole tumor (WT)	Swin UNETR	0.9351 ± 0.0026	0.8774 ± 0.0032	0.9980 ± 0.0003	0.9403 ± 0.0028	0.9273 ± 0.0030
	SegResNet	0.9125 ± 0.0030	0.8390 ± 0.0037	0.9972 ± 0.0004	0.8947 ± 0.0032	0.9289 ± 0.0031
Tumor core (TC)	Swin UNETR	0.9009 ± 0.0034	0.8195 ± 0.0041	0.9989 ± 0.0002	0.8879 ± 0.0035	0.9171 ± 0.0033
	SegResNet	0.9106 ± 0.0032	0.8362 ± 0.0039	0.9991 ± 0.0002	0.9253 ± 0.0033	0.8969 ± 0.0036
Enhancing tumor (ET)	Swin UNETR	0.8945 ± 0.0042	0.8096 ± 0.0050	0.9993 ± 0.0001	0.9021 ± 0.0039	0.8876 ± 0.0042
	SegResNet	0.8328 ± 0.0048	0.7132 ± 0.0056	0.9987 ± 0.0003	0.8153 ± 0.0043	0.8515 ± 0.0046

Interestingly, the metrics in TC region presents a competitive scenario. While Swin UNETR maintains a high mean Dice score (0.9009), SegResNet slightly outperforms it with a higher mean Dice score (0.9106), better precision and better Accuracy. This indicates that while Swin UNETR backbone is better at overall overlap, SegResNet backbone may be slightly more “conservative” and precise in identifying the core boundaries.

The ET region, which is often the most challenging due to its smaller size and irregular morphology, shows the widest performance gap. Swin UNETR achieves a mean Dice of 0.8945, which is significantly higher than SegResNet’s 0.8328. The IoU drop for SegResNet to 0.7132 indicates the difficulty faced by CNN based Segresnet backbone with fine-grained segmentation in this sub-region while Swin Transformer provide a better performance due to its hierarchical feature representation.

Across all regions, exceptionally high Accuracy (all >0.997) with minimal standard deviation is observed, which is expected in medical segmentation due to the heavy imbalance of background pixels versus the target ROI. However, the Dice and IoU metrics provide a more realistic view of the segmentation quality.

The high Sensitivity scores ranging from 0.85 to 0.92 with minimal variation across both models indicate their strong capability to avoid false negatives which is a very critical factor in clinical settings to ensure no part of the tumor is overlooked. Comparing between the pretrained model backbones, Swin UNETR provides a better balance between Precision and Sensitivity, whereas SegResNet shows more fluctuation, particularly in the challenging ET region.

### 5.3 Component-wise ablation of ensemble strategies

Building upon the baseline performance of the individual models, this section presents a component wise ablation of the successive ensemble mechanisms quantify the increments in contribution due to each design choice.

As discussed in Section V-B, Swin UNETR is found to excel in global structure (WT and ET) while Segresnet shows strong performance in the core (TC) with a high precision. This variance in performance strengths in different tumor regions between the two models suggests that ensembling is highly viable for this dataset. By combining the transformer’s global contextual learning with the convolutional model’s local spatial precision, an ensemble is likely to smooth out the individual model errors and provide a more robust and generalized segmentation output (Table 2).

**Table 2:** Per region Dice scores for ensemble strategies (mean ± SD over three independent runs).

Strategy	Whole tumor (WT)	Tumor core (TC)	Enhancing tumor (ET)
Simple averaging (SA)	0.9352 ± 0.0028	0.9113 ± 0.0039	0.8820 ± 0.0051
Region adaptive weighting (RSW)	0.9405 ± 0.0019	0.9183 ± 0.0031	0.9068 ± 0.0044
Disagreement-based adaptive	0.9447 ± 0.0021	0.9231 ± 0.0033	0.9071 ± 0.0041

The component-wise ablation results demonstrate a clear progressive improvement in segmentation performance as successive ensemble mechanisms are added.

#### 5.3.1 Simple averaging (SA)

The SA strategy provides a foundational baseline, slightly improving upon the best individual model scores in WT and TC regions. However, the dice score in ET region is slightly reduced compared to Swin UNETR’s score probably because the strategy considers both the models equally capable. This strategy mitigates the risk of a single model’s catastrophic failure but fails to capitalize on the specific regional strengths of each of the models that was identified earlier.

#### 5.3.2 Region-adaptive soft-weighting (RSW)

The RSW strategy shows a noticeable performance boost over SA for all regions and particularly in the Enhancing Tumor (ET) region, which achieved a dice score of 0.9064 outperforming the individual model scores. This confirms that assigning weights based on prior validation performance of individual models allows the ensemble to lean more heavily toward the model that have better strength in that particular region.

For each independent run, the weights for RSW are calculated by normalizing the validation Dice scores obtained for each model in each tumor sub-region in that particular run.

For illustrative purposes, the approximate weights can be calculated using the mean dice values obtained in Table 1, although in practice weights are computed per run.

For Whole Tumor (WT) region,


$$w_{\left\{\text{Swin},\mathrm{WT}\right\}}=\frac{0.9351}{0.9351+0.9125}\approx 0.506$$

$$w_{\left\{\text{SegResNet},\mathrm{WT}\right\}}=\frac{0.9122}{0.9351+0.9125}\approx 0.494$$

For Tumor Core (TC) region,


$$w_{\left\{\text{Swin},\mathrm{TC}\right\}}=\frac{0.9009}{0.9009+0.9106\;}\approx 0.497$$

$$w_{\left\{\text{SegResNet},\mathrm{TC}\right\}}=\frac{0.9106}{0.9009+0.9106}\approx 0.503$$

For Enhancing Tumor (ET) region,


$$w_{\left\{\text{Swin},\mathrm{ET}\right\}}=\frac{0.8945}{0.8945+0.8328}\approx 0.518$$

$$w_{\left\{\text{SegResNet},\mathrm{ET}\right\}}=\frac{0.8328}{0.8945+0.8328}\approx 0.482$$

These weights slightly favour Swin UNETR outputs for WT and ET and Segresnet assumptions for TC region, therefore making better utilization of individual models’ production based on their performance. While the region-specific weighted (RSW) ensemble improves overall performance compared to simple averaging, it does not explicitly account for regions with high prediction uncertainty, such as tumor boundaries where the predictions of SegResNet and Swin UNETR can differ substantially.

#### 5.3.3 Disagreement-based region specific ensembling (DRE)

The proposed Disagreement-based refinement addition to Region Adaptive Soft Weighting achieves the best scores across the board, with a peak WT Dice of 0.9447, TC Dice of 0.9231 and ET Dice of 0.9071. By analyzing the level of disagreement between the individual models, this method dynamically switches between the weighted approach and confidence-based approach. In the regions where the disagreement between the model predictions were low, such as the inner areas of tumor, the weighted average similar to RSW ensemble gave accurate results. But in the areas of high uncertainty with disagreement (d) exceeds the threshold (𝜏 = 0.5), particularly in the region boundaries, the prediction of a more confident model was taken which would have resulted in better validation scores compared to the previous methods.

To further analyze the DRE mechanism, we calculated the fraction of voxels with d (v,c) ≥ *τ* = 0.5 across the 56 test samples that triggered the confidence-based selection branch. Averaging across three runs, 4.8% ± 0.6% of voxels activated the disagreement branch and those were concentrated mostly around tumor boundary regions, particularly the WT–TC and TC–ET interfaces. In these boundary voxels, DRE changed the final label relative to RSW in 31.4% ± 3.2% of cases, with the change being correct (i.e., matching ground truth) in 68.7% ± 2.8% of those cases leading to a marginal improvement in segmentation results.

Overall, the validation metrics suggest that the disagreement-based weighted ensemble is the most viable path 3D MRI segmentation of brain tumor. This proposed method successfully recovers some fine details in previously lost in the region boundaries leading to better validation metrics and proving that dynamic region specific weighting brings some improvements over static averaging. Furthermore, the statistical significance of these improvements is confirmed by a Wilcoxon signed-rank tests in the next section.

### 5.4 Statistical significance analysis

To validate that the performance improvements of the Disagreement based ensemble are statistically, we conducted pairwise Wilcoxon signed-rank tests where its per-case Dice scores were compared against the individual models and two ensemble architectures. Here, wilcoxon signed-rank test was chosen over a paired t-test as it makes no normality assumption on the distribution of Dice score differences, which is appropriate given the skewed distribution of ET scores at the tail of the performance range. All tests are two-sided with a significance threshold of *α* = 0.05. The reported test statistic (W) is the Wilcoxon signed-rank statistic, which is the minimum of the sums of positive and negative ranks. Full results are presented in Table 3.

**Table 3:** Wilcoxon signed-rank test results: DRE ensemble vs baseline methods (*n* = 56).

Comparison against	Region	Test statistic	*p*-value
SegResNet	WT	60.0	< 0.001
	TC	426.0	0.0024
	ET	81.0	< 0.001
Swin UNETR	WT	378.0	0.0006
	TC	233.0	< 0.001
	ET	604.0	0.1135
Simple Averaging Ensemble (SA)	WT	360.0	0.0004
	TC	504.0	0.0165
	ET	280.0	< 0.001
Region Adaptive Weighting Ensemble (RSW)	WT	555.0	0.0475
	TC	670.0	0.2964
	ET	751.0	0.7014

#### 5.4.1 Disagreement ensemble vs. individual models

The disagreement-based ensemble shows statistically significant improvements over SegResNet in all three regions, indicating that this ensemble outperforms the CNN baseline across all the tumor regions. Against Swin UNETR, it achieves significant improvements in WT and TC but in ET it does not reach statistical significance, which is expected as Swin UNETR is already has strong performance in ET and the ensemble’s gain of +0.0121 dice over Swin UNETR in ET is less significant due to higher variance in ET scores and limited sample size.

#### 5.4.2 Disagreement ensemble vs. ensemble baselines

Against SA, the disagreement-based ensemble shows significant improvements across all three tumor regions, indicating the benefit of dynamic fusion basic averaging. The strongest margin is in ET, where static averaging might have suppressed some correct predictions in ambiguous boundary voxel. Against RSW, the disagreement-based ensemble achieves significance only in WT while TC and ET comparisons are less significant. This reflects the incremental rather than transformative nature of the disagreement-based ensemble over region-adaptive weighting alone. In regions where the RSW weights already improve the performance (TC and ET), the additional disagreement-based fusion only adds a marginal benefit that does not rise to statistical significance on this test set size. This is consistent with the ablation results in Section V-C which shows that the primary performance driver is region-adaptive weighting, while the disagreement-based fusion contributes an incremental gain primarily in WT boundary regions.

These results support the claim that the proposed disagreement-based ensemble achieves a statistically reliable improvement over both the individual models as well as the simple averaging baseline, while we also acknowledge that the marginal gains over RSW in individual sub-regions require validation on a larger test set.

### 5.5 Output analysis

**Figure 10:**
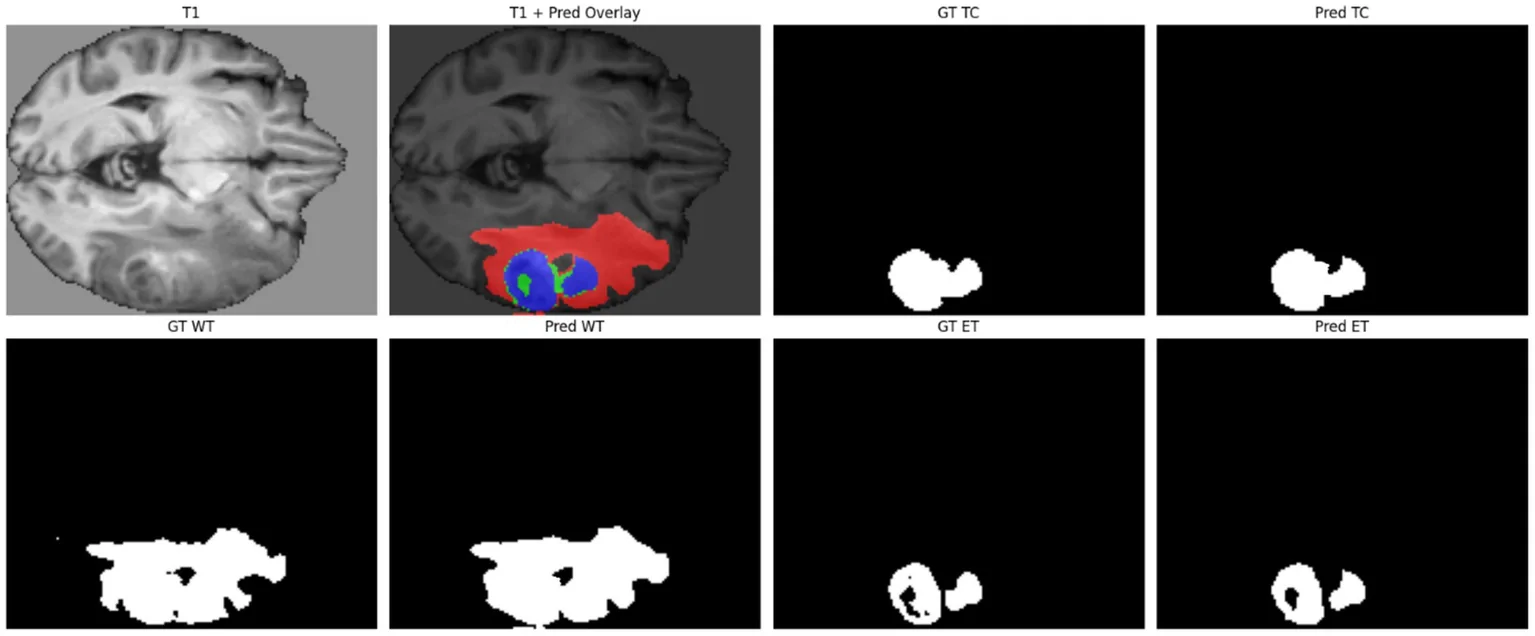
Sample prediction output for SegResNet (red = WT, green = TC, purple = ET).

**Figure 11:**
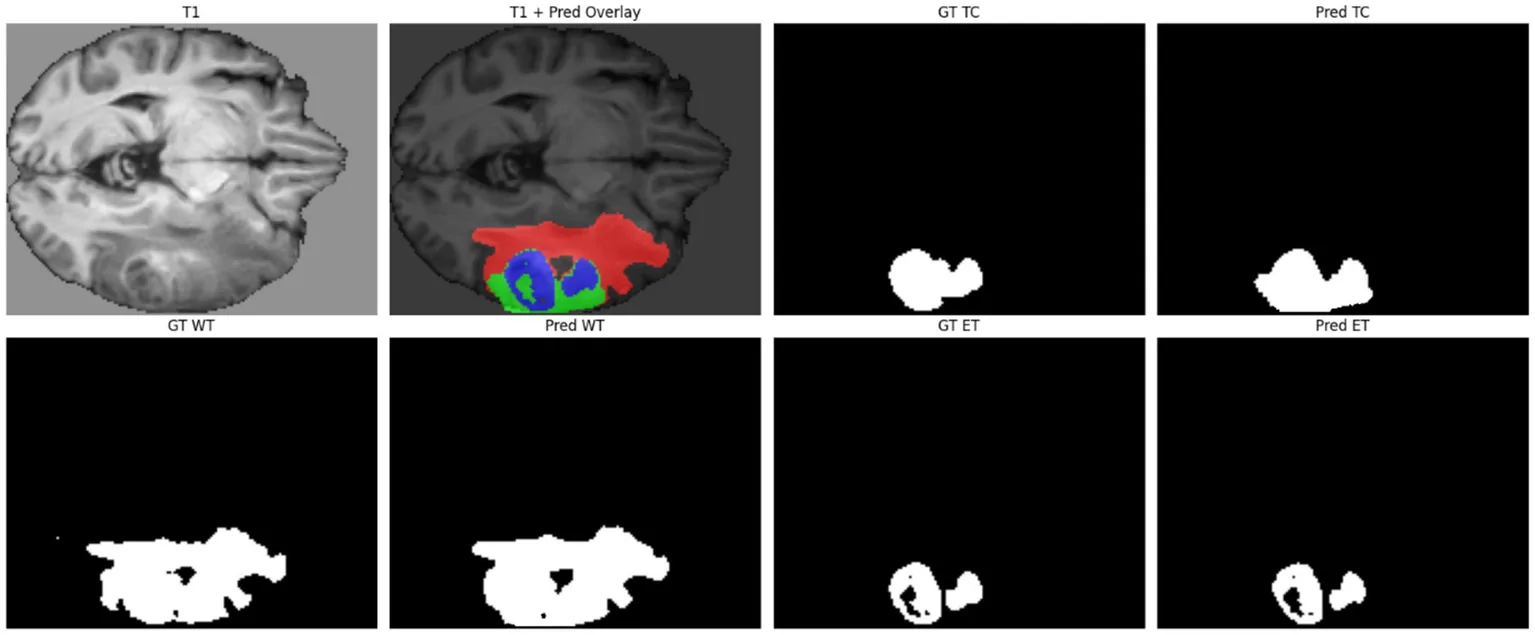
Sample prediction output for Swin UNETR (Red = WT, Green = TC, Purple = ET).

**Figure 12:**
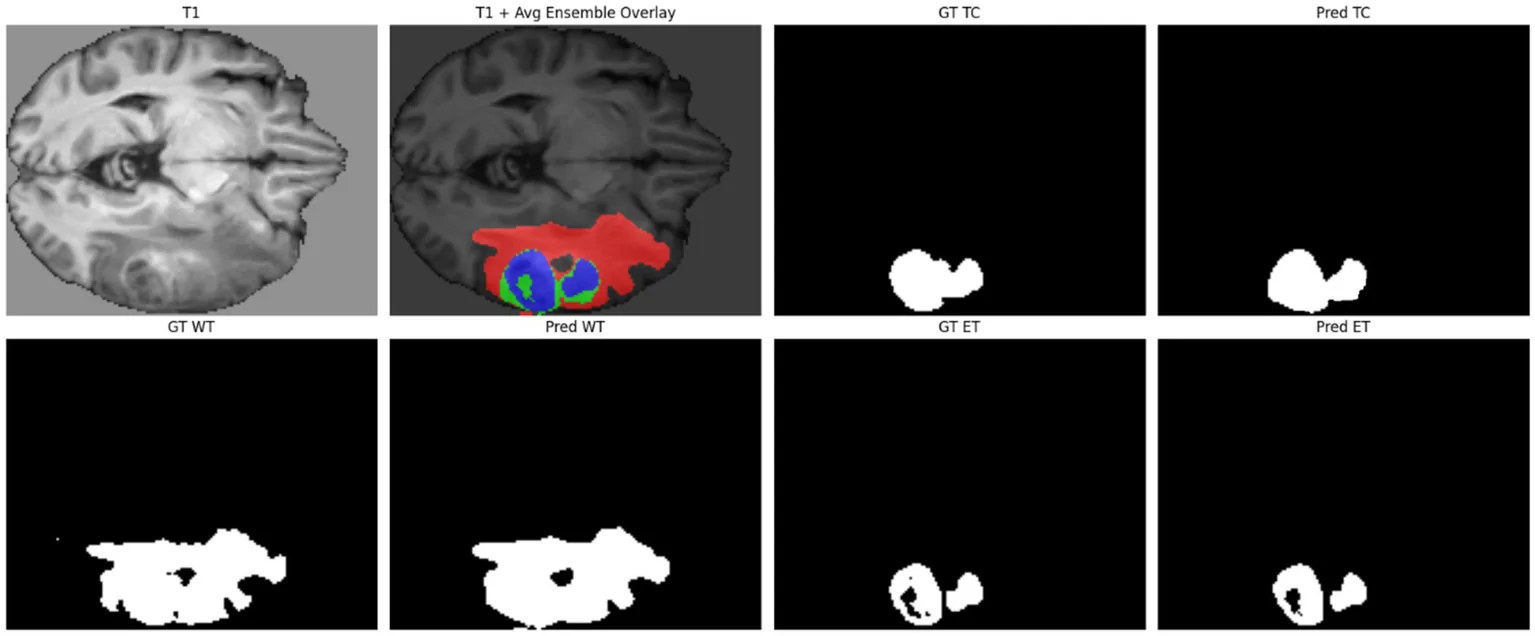
Sample prediction output for simple average ensemble (red = WT, green = TC, purple = ET).

**Figure 13:**
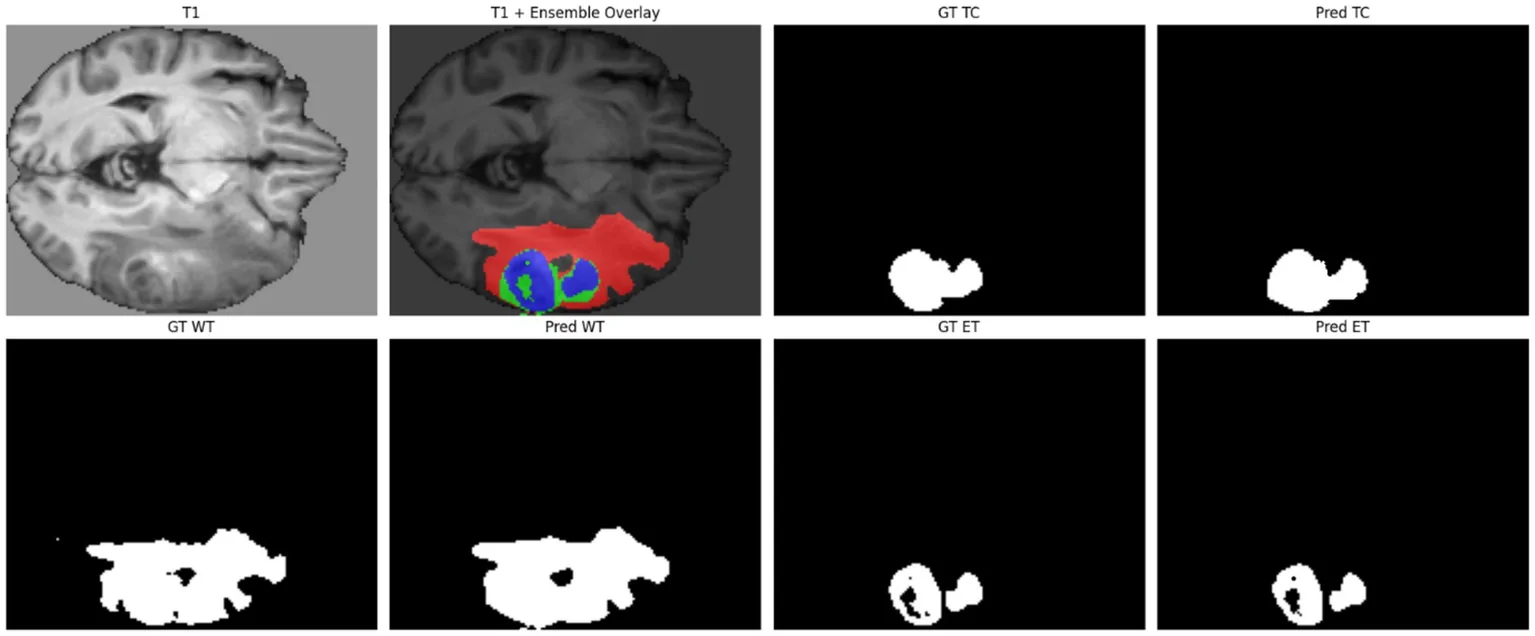
Sample prediction output for Region Adaptive Soft Weighting Ensemble (red = WT, green = TC, purple = ET).

**Figure 14:**
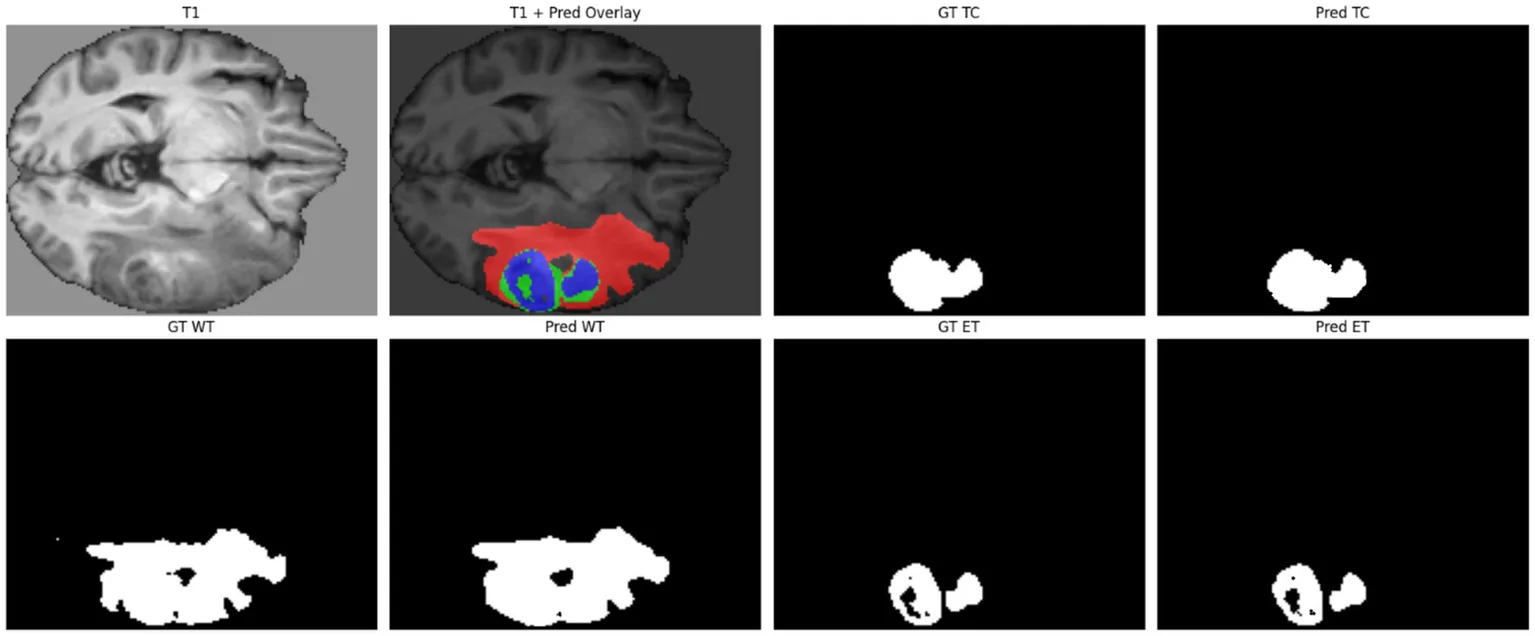
Sample prediction output for disagreement based region adaptive ensemble. (Red = WT, Green = TC, Purple = ET).

The individual models as well as the ensemble methods are able to generate impressive and near accurate segmentation outputs as we can see from Figures 10–14.

Figures 10, 11 present the segmentation outputs and the corresponding ground truths for SegResNet and Swin UNETR, respectively. Upon analyzing the segmentation outputs of individual models, the predicted regions are consistently close to the ground truth (GT). While both models perform well in the Whole Tumor (WT) region, SegResNet exhibits higher accuracy at the segmentation boundaries. In contrast, Swin UNETR outperforms SegResNet in the Enhancing Tumor (ET) region, specifically in its ability to segment minute sections that likely contributed to its superior evaluation metrics. However, SegResNet notably outperforms Swin UNETR within the Tumor Core (TC) region.

Figures 12–14 present the segmentation outputs by the ensemble techniques and the corresponding ground truths. The ensemble techniques yield superior results by effectively combining the strengths of both architectures. The Simple Average (SA) ensemble compensates for Swin UNETR’s limitations in the TC region by incorporating SegResNet’s specialized performance there. This segmentation is further refined in the weighted ensemble, which biases the output toward SegResNet’s TC predictions; ultimately, the disagreement-based ensembling approach achieves a result nearly identical to the ground truth.

In the WT region, the inaccurate segmentation produced by SegResNet at the lower boundary persists in the Simple Average output. While the weighted ensemble favoring Swin’s predictions reduces this error, the disagreement-based model eliminates the inaccuracy entirely. This is likely because the technique identifies high disagreement between models and prioritizes the more confident output, in this case, that of Swin UNETR.

For the ET region, the Simple Average ensemble outperforms SegResNet but remains slightly less accurate than the standalone Swin UNETR results. The weighted ensemble provides a more robust output by biasing toward Swin’s performance. Finally, the disagreement-based ensemble provides the closest alignment to the ground truth, as it preserves even the smallest accurately segmented regions identified by the individual models. Overall, we could see that disagreement-based adaptive ensemble is able to effectively combine the complementary confidence and strength of both models and suppress their individual weaknesses with greater accuracy compared to the other techniques.

### 5.6 Comparative analysis

Table 4 presents a comparative analysis of the proposed ensemble strategies against state-of-the-art methods from literature. Methods evaluated on BraTS 2020 are directly comparable to the proposed approach whereas the methods from different BraTS challenge years or different datasets are provided for contextual reference.

**Table 4:** Comparative analysis with established architectures.

Study	Dataset	WT	TC	ET	Mean
CNN with small 3 × 3 kernels, intensity normalization, and data augmentation (Pereira et al., 2016)	BRATS 2013 & BRATS 2015	0.88	0.83	0.77	0.826
FCN with ResNet50 (RGB fusion of T1w, T2w, and average) (Lu et al., 2025)	Custom clinical dataset (203 subjects)	NA	NA	NA	0.937
2D U-Net with three convolutional layers per block (Cherguif et al., 2019)	BRATS 2017	0.81	NA	NA	NA
Federated Learning (FL) using a U-Net topology (Sheller et al., 2019)	BraTS 2018 (High-Grade Glioma, FLAIR modality)	NA	NA	NA	0.852
SAC UW-Net (Self-attention blocks) (Pal et al., 2024)	BraTS 2020	NA	NA	NA	0.884
(Purohit and Bhatt, 2025) Review of Deep Learning architectures (CNN, U-Net, Transformers) and Optimizers	Synthesis of BraTS (2018, 2020, 2021) and public datasets	0.91	0.85	0.84	0.866
M-Net (Mesh-Cast mechanism with Two-Phase Sequential (TPS) training) (Lu et al., 2023)	BraTS 2019	0.8838	0.9052	0.9143	0.9011
DUCK-U-Net (Fusion of DUCK-Net and traditional U-Net) (Majumdar et al., 2025)	BraTS 2020 (First 50 cases)	NA	NA	NA	0.7491
Ensemble of 3D U-Nets (Voxel-wise averaging) (Feng et al., 2020)	BraTS 2018	0.926	0.911	0.870	0.9023
Attention-based U-Net (Zhou and Zhu, 2023)	BraTS 2018	0.861	0.871	0.789	0.84
Disentangled Fusion Network (Zhou et al., 2026)	BraTS 2020	0.879	0.895	0.826	0.827
SegResNet	BraTS 2020	0.9125	0.9106	0.8328	0.8853
Swin UNETR	BraTS 2020	0.9351	0.9009	0.8945	0.9102
Simple average ensemble (SA)	BraTS 2020	0.9352	0.9113	0.8820	0.9095
Region adaptive soft weighted ensemble (RSW)	BraTS 2020	0.9405	0.9183	0.9068	0.9219
Disagreement-based adaptive fusion ensemble (proposed)	BraTS 2020	0.9447	0.9231	0.9071	0.9249

The model backbones achieve excellent evaluation results across the tumor sub-regions. Our individual models, Swin UNETR (0.9102 Mean Dice) and SegResNet (0.8853 Mean Dice) achieved competitive metrics when compared to few established methods like DUCK-U-Net and Federated Learning which were also evaluated on BraTS 2020 dataset. This indicates that our backbone models are strong and provide robust support for the overall architecture to effectively capture the spatial complexities required for accurate tumor segmentation.

In addition to conventional single-model architectures, fusion based approaches such as attention-based U-Net (Zhou and Zhu, 2023) and disentangled fusion network (Zhou et al., 2026) have improved performance by estimating uncertainty during feature fusion. As reflected in Table 4, they achieve high Dice Scores across tumor sub-regions but their performance remains limited particularly in the ET region which could be due to model bias toward dominant region as a result of class imbalance in BraTS dataset. Also, as they rely on single model architecture, this absence of model level diversity restricts their ability to consistently handle the heterogeneity and imbalance present across tumor sub-regions. The M-Net (Lu et al., 2023) architecture performs extremely well in the ET and TC region probably due to its strength to capture very fine-grained features, resulting to a mean dice of 0.9011. Additionally, the ensemble of 3D U-Nets in (Feng et al., 2020) also has achieved high dice scores across all tumor sub-regions and with significant scores in ET region. Although direct numerical comparison with our models is not valid due to difference in evaluation setups, still it can be noticed that our individual model backbones seem to lack this level of consistent performance across tumor regions as observed in fusion based architectures, potentially due to their respective inductive biases toward local feature modeling and global contextual representation.

Upon combining SegResNet’s ability to capture fine-grained details with Swin UNETR’s global contextual knowledge, our ensemble architectures were able to provide a better and consistent result across all regions and resulting to higher Dice scores than individual models. Out of all of them, the disagreement-based ensemble was able to achieve best evaluation results with an improved mean dice of 0.9249 due to its ability to address individual weaknesses of models and combining their strengths in a dynamic manner. Overall, the results show that this approach produces competitive metrics when compared to current state-of-the-art architectures and outperforms the backbone model performance, performance of static ensembling methods in this study as well as other methods that were evaluated in BraTS 2020. However, a fair dice score based comparison with other methods would require evaluation over a common dataset with a common evaluation setup which we leave for future scope of this research.

### 5.7 Sensitivity analysis of disagreement threshold

**Figure 15:**
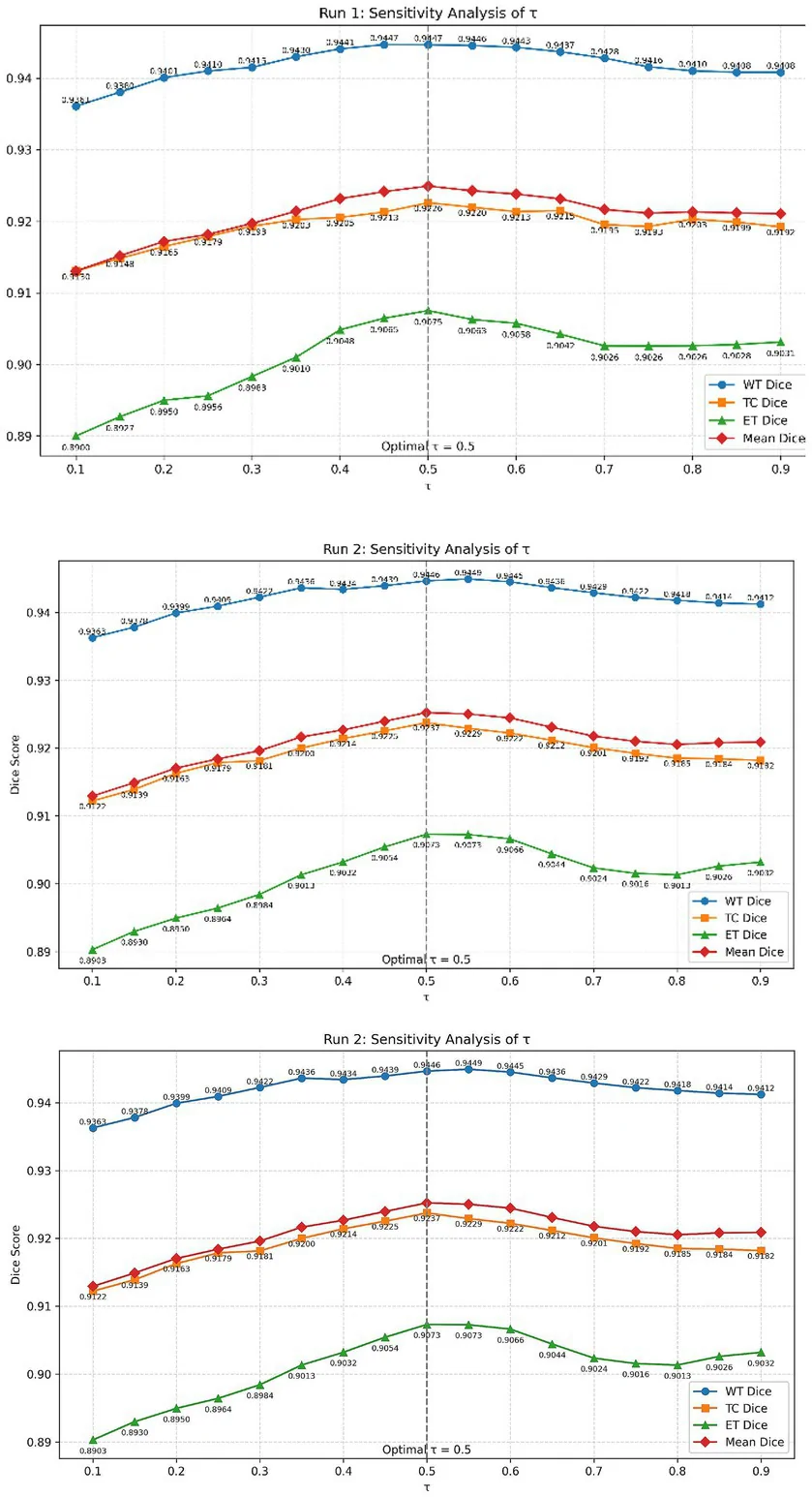
Sensitivity analysis of disagreement threshold (*τ*) where *τ* = 0.5 yields the best overall performance across all 3 runs.

The disagreement threshold *τ* is responsible for the switch in the behavior of the proposed Disagreement-based Fusion Ensemble between the region-adaptive soft weighting and confidence-based model selection. To evaluate the robustness of the architecture and justify the choice of *τ*, a sensitivity analysis was conducted over the range τ ∈ [0.1, 0.9] with uniform increments of Δτ = 0.05 against a new random set of 60 samples (excluding test set) for three independent runs as illustrated in the line graphs in Figure 15. For each run, the dice sensitivity trend shows that WT demonstrates high stability, TC shows moderate stability and ET shows slightly higher variability possibly due to its boundary dominant morphology. The consistency of the dice sensitivity trend across all the runs demonstrates the reproducibility of the framework with respect to different random initializations.

At lower threshold values (*τ* = 0.1–0.3), even small disagreements between SegResNet and Swin UNETR predictions are classified as high uncertainty due to which ensemble frequently follows confidence based single model selection instead of fusion of complementary model strengths reducing overall cooperation during classification.

The higher threshold values (τ = 0.7–0.9) lead to a relatively conservative ensemble in terms of detecting any disagreement. This reduces overall boundary refinement causing decline to segmentation accuracy.

The mid-range threshold values (τ = 0.4–0.6) achieve most effective balance between region-specific soft-weighting approach and confidence-based selection. For each of the runs, peak mean dice score was consistently observed at τ = 0.5, which confirms that τ = 0.5 provides the best trade-off between confidence-based selection and cooperative region-adaptive fusion. This selection also aligns theoretically with nature of sigmoid outputs that generally center around 0.5.

In this study, we used a single global τ=0.5 across WT, TC, and ET for simplicity and to avoid overfitting to a limited dataset of 56 samples. Also, the trends in Figure 15 suggest similar mid-range optima around τ=0.5 across regions, but we do acknowledge that region-specific thresholds could further improve performance and leave this for future work.

### 5.8 Generalizability analysis: zero-SHOT evaluation on BRATS 2021

To provide an preliminary pilot assessment to generalizability of the architecture beyond BraTS2020 a zero-shot evaluation was conducted for the three ensemble strategies. For this a random sample of 50 patient cases (32 high grade and 18 low grade) was taken from BraTS 2020. BraTS 2021 shares same four modality (T1, T1ce, T2, FLAIR) and annotation (WT, TC and ET) as BraTS2020 that enables direct metric comparison. No re-training or finetuning was performed for this evaluation.

The evaluation results are presented in Table 5 where some drop in performance is seen as expected in a zero-shot evaluation. But, the relative ordering of performance between the strategies is still fully preserved, indicating the performance advantage of disagreement based adaptive over other strategies irrespective of dataset. However, it must be emphasized that this 50-sample zero-shot evaluation should not be considered as the performance benchmark of this architecture on BraTS 2021 as the ensemble would improve further when the individual models are explicitly finetuned on BraTS 2021.

**Table 5:** Zero shot evaluation results in BraTS2021.

Strategy	Whole tumor (WT)	Tumor core (TC)	Enhancing tumor (ET)	Mean
Simple averaging (SA)	0.9184	0.8891	0.8532	0.8869
Region adaptive weighting (RSW)	0.9258	0.8983	0.8791	0.9011
Disagreement-based adaptive (DRE)	**0.9301**	**0.9047**	**0.8864**	**0.9071**

The bold values indicates the Disagreement-based Fusion approach to this problem was shown to be superior to either the Simple Average or the Region Adaptive Soft Weighting methods.

## 6 Limitations and future scope

While the proposed Disagreement-based ensemble technique demonstrates an improvement in the segmentation results, there were some constraints and limitations in this study that need further refinement and validation before any professional adaptation.

Firstly, the training and evaluation in this study was done on a subset of BraTS 2020 dataset with total 368 samples (257 training, 55 validations, 56 test), particularly due to massive resource requirements for 3D MRI volumes processing and limited compute and memory available to us. While the architecture was able to show promising results, this may not fully cover the diverse morphological variations and imaging artifacts in broader clinical populations. Also due to hardware limitations in GPU and RAM, the individual models were trained for a limited number of iterations with limited batch size, i.e., 65 epochs for SegResNet and 80 epochs for Swin UNETR. This suggests the base models might not have fully reached their global minima potentially limiting the maximum achievable performance during the ensemble.

While finetuning Swin UNETR we entirely froze the patch embedding layer and early encoder stages as in our earlier experiments, fully unfreezing the early layers resulted in slightly lower segmentation accuracy, whereas retaining the pretrained initialization in the early stages yielded marginally better results. We believe this is likely because excessive modification of low-level pretrained feature representations can reduce the benefit of transfer learning, particularly in case of small sized training dataset. Thus, a more systematic investigation can be done for fine-tuning depth, layer-wise adaptation strategies, and domain adaptation techniques while fine tuning the models over a larger dataset.

Also in this study, the disagreement threshold was selected through a systematic sensitivity analysis conducted over the range *τ* ∈ [0.1, 0.9] with uniform increments of Δτ = 0.05. This might be effective for an initial proof-of-concept but this static threshold value might not be able to encounter the varying noise levels and contrast differences in larger MRI datasets. Future iterations of this work should therefore focus on more fine-grained threshold optimization with Δ*τ* ≪ 0.05 over the full continuous range (0< 𝜏 <1). Additionally, an adaptive threshold selection mechanism could be explored to dynamically adjust the disagreement threshold based on local image statistics and model confidence distributions rather than relying on a fixed global value.

The current formulation has a limitation that the independent WT/TC/ET prediction does not inherently enforce the fact that these regions follow a nested hierarchy ET ⊆ TC ⊆ WT. Adding an explicit post-processing step or a structural constraint here would improve the anatomical consistency and clinical interpretability of the predictions. Also, the ensemble requires inference from two independent 3D backbones with SegResNet (~4 M parameters) and Swin UNETR (~16 M parameters) which increases the computational overhead during training and inference. Future works need to analyze the cost–benefit tradeoff between a hybrid ensemble or light weight alternatives to reduce any overhead in deployment.

While the three-run variance analysis presents the reproducibility of the architecture, a cross-dataset generalizability still remains an open question. The zero-shot evaluation on BraTS 2021 was conducted without any retraining, i.e., the region confidence scores derived from BraTS 2020 validation and the disagreement threshold τ were transferred directly to BraTS 2021 inference without any adaptation. The results from zero-shot evaluation indicate that the disagreement-based ensemble retains its performance advantage in this setting. However, this does not constitute a complete assessment of generalizability and the absolute scores on BraTS 2021 are expected to improve with a dedicated fine-tuning. In addition to that we recommend full external validation of the proposed architecture beyond BraTS 2020/21 to the future works along with failure case analyses and disagreement visualization maps.

Moving beyond Swin UNETR and SegResNet, the framework could also be extended to include other state-of-the-art architectures, such as nnU-Net or Vision Transformers (ViT). This addition of diversity in the model pool would likely enhance the ensemble’s ability to mitigate individual architectural biases.

To further ensure the robustness of this disagreement-based logic, it is essential to benchmark this method against multiple large-scale datasets such as complete set of BraTS datasets, Medical Segmentation Decathlon, etc. This would validate the model’s performance across different scanner types and institutional protocols.

Moving toward clinical deployment, the ensemble technique could be made more dynamic by incorporating Active Learning or Test-Time Augmentation (TTA). The robustness of the architecture was evaluated via synthetic augmentation based noise to simulate scanner variabilities, but this needs to be further strengthened by explicitly training and evaluating the models against multi scanner data with real world variation and noise. Additionally, a formal calibration analysis needs to be done with reliability diagrams and expected calibration errors to more rigorously assess reliability of the architecture alongside Dice and IoU.metrics. Such advancements would allow the ensemble to adapt to real-life medical data, where image quality and tumor presentation can vary significantly from curated research datasets.

## 7 Conclusion

The ability of the proposed Disagreement-based ensemble method to perform automated segmentation of brain tumors using 3D MRI data was supported by research demonstrating its effectiveness. This ability is the result of combining both the global information provided by Swin UNETR and the local spatial information of SegResNet to overcome the model limitations associated with each. This study demonstrated that while Swin UNETR performed better when assessing enhancing tumor, and SegResNet performed better when assessing tumor core, neither of these results was sufficient.

The Disagreement-based Fusion approach to this problem was shown to be superior to either the Simple Average or the Region Adaptive Soft Weighting methods. The results of the Disagreement-based Fusion technique achieved consistent dice scores of WT: 0.9447, TC: 0.9231, and ET: 0.9071 with minimum variance across 3 independent runs, i.e., maximum for the Whole Tumor region and a significant increase in accuracy in the much more difficult ET region.

Though the study was carried out with some limitations in resources, such as the size of the dataset and the number of training epochs, the qualitative and quantitative improvements achieved in the process, three-independent-run training strategy, statistical validation and zero-shot generalizability to BraTS2 2021 collectively lay a strong foundation for future development. In future, the extension of the methodology to larger, more diverse analysis and training across datasets, along with the optimization of the disagreement framework, is a major step toward a more generalized and clinically deployable tool in the field of neuro-oncology.

## Data Availability

The original contributions presented in the study are included in the article/supplementary material, further inquiries can be directed to the corresponding author.
